# The activation of iron deficiency responses of grapevine rootstocks is dependent to the availability of the nitrogen forms

**DOI:** 10.1186/s12870-024-04906-y

**Published:** 2024-03-26

**Authors:** Sarhan Khalil, Rebeka Strah, Arianna Lodovici, Petr Vojta, Federica De Berardinis, Jörg Ziegler, Maruša Pompe Novak, Laura Zanin, Nicola Tomasi, Astrid Forneck, Michaela Griesser

**Affiliations:** 1 University of Natural Resources and Life Sciences, Vienna, Department of Crop Sciences, Institute of Viticulture and Pomology, Tulln an der Donau, Austria; 2 National Institute of Biology, Department of Biotechnology and Systems Biology, Ljubljana,, Slovenia; 3 Jožef Stefan International Postgraduate School, Ljubljana, Slovenia; 4 University of Udine, Department of Agricultural, Food, Environmental, and Animal Sciences, Udine, Italy; 5 University of Natural Resources and Life Sciences, Vienna, Department of Biotechnology, Institute of Computational Biology, Vienna, Austria; 6 Leibniz Institute of Plant Biochemistry, Department Molecular Signal Processing, Halle (Saale), Germany; 7 University of Nova Gorica, Faculty of Viticulture and Enology, Vipava, Slovenia

**Keywords:** Vitis, Iron uptake, Chlorosis, Nitrate, Ammonium, RNA-Seq

## Abstract

**Background:**

In viticulture, iron (Fe) chlorosis is a common abiotic stress that impairs plant development and leads to yield and quality losses. Under low availability of the metal, the applied N form (nitrate and ammonium) can play a role in promoting or mitigating Fe deficiency stresses. However, the processes involved are not clear in grapevine. Therefore, the aim of this study was to investigate the response of two grapevine rootstocks to the interaction between N forms and Fe uptake. This process was evaluated in a hydroponic experiment using two ungrafted grapevine rootstocks Fercal *(Vitis berlandieri* x *V. vinifera*) tolerant to deficiency induced Fe chlorosis and Couderc 3309 (*V. riparia* x *V. rupestris*) susceptible to deficiency induced Fe chlorosis.

**Results:**

The results could differentiate Fe deficiency effects, N-forms effects, and rootstock effects. Interveinal chlorosis of young leaves appeared earlier on 3309 C from the second week of treatment with NO_3_^−^/NH_4_^+^ (1:0)/-Fe, while Fercal leaves showed less severe symptoms after four weeks of treatment, corresponding to decreased chlorophyll concentrations lowered by 75% in 3309 C and 57% in Fercal. Ferric chelate reductase (FCR) activity was by trend enhanced under Fe deficiency in Fercal with both N combinations, whereas 3309 C showed an increase in FCR activity under Fe deficiency only with NO_3_^−^/NH_4_^+^ (1:1) treatment. With the transcriptome analysis, Gene Ontology (GO) revealed multiple biological processes and molecular functions that were significantly regulated in grapevine rootstocks under Fe-deficient conditions, with more genes regulated in Fercal responses, especially when both forms of N were supplied. Furthermore, the expression of genes involved in the auxin and abscisic acid metabolic pathways was markedly increased by the equal supply of both forms of N under Fe deficiency conditions. In addition, changes in the expression of genes related to Fe uptake, regulation, and transport reflected the different responses of the two grapevine rootstocks to different N forms.

**Conclusions:**

Results show a clear contribution of N forms to the response of the two grapevine rootstocks under Fe deficiency, highlighting the importance of providing both N forms (nitrate and ammonium) in an appropriate ratio in order to ease the rootstock responses to Fe deficiency.

**Supplementary Information:**

The online version contains supplementary material available at 10.1186/s12870-024-04906-y.

## Introduction

Iron (Fe) is considered a key micronutrient in plants, as its active form is involved in many biological processes, such as chlorophyll synthesis, photosynthesis, respiration, oxidative stress responses, and nitrogen (N) assimilation [1, 2]. In the earth’s crust, Fe is the fourth most abundant element, mostly present in the soil solution in ferric (Fe^3+^) form, while only the ferrous form (Fe^2+^) can be used by non-graminaceous plants [3]. The low solubility of this micronutrient in soil solution limits its uptake by plants, particularly in alkaline and calcareous soils as they are characterized by high pH values [4]. Any limitation in Fe availability will affect plant development leading to reduced yield and quality losses [5, 6]. The main symptoms of Fe deficiency in grapevine appear first on young leaves as interveinal chlorosis (yellow color of interveinal areas with main vines remaining green) which can develop necrosis spots on the blade under prolonged conditions of Fe starvation [7].

Grapevine rootstocks have been characterized to have different abilities for taking up and transporting nutrients, as well as regulating hormones and signaling molecules under different stresses. Therefore, using properly selected rootstocks could be a cost-effective and efficient way to prevent Fe chlorosis [8]. . Rootstocks resulting from the crossbreeding between *Vitis berlandieri* (Planch.) and *V. vinifera* (L.) (e.g., Fercal and 41B) have been classified as tolerant to Fe chlorosis. In contrast, the rootstocks from crossbreeding between *V. riparia* (Michx.) and *V. rupestris* (Scheele) (e.g., 3309 C and 101 − 14 Mgt) have been considered highly susceptible to Fe chlorosis [9–11]. Plants have evolved mechanisms or structural features to maintain Fe balance in the plant tissue when grown on Fe-deficient soils. Different physiological as well as morphological changes may occur in response to low Fe availability [12, 13], such as an increase in the number of lateral roots [14, 15], or an enhanced ability to acidify the rhizosphere to increase Fe availability, and an induction of the ferric chelate reductase (FCR) activity [16, 17].

In plants, nitrogen (N) is crucial for the synthesis of proteins, nucleic acids, and chlorophyll [18]. Plants can use both, nitrate (NO_3_^−^) and ammonium (NH_4_^+^), but the availability of each form and the ratio to each other directly influences the uptake of other nutrients and the pH of the rhizosphere, as well as many plant physiological and biochemical processes, and consequently plant growth and development [19–21]. Nitrate (NO_3_^−^) is the preferred N source applied to fruit trees cultivation, especially for grapevines [22], but high NO_3_^−^ levels are a risk factor for Fe chlorosis as the pH around roots elevates [23] and the Fe transport from roots to shoots is inhibited [24, 25]. On the other hand, ammonium (NH_4_^+^) application could have a positive effect on nutrient absorption, which has been shown in grapevine where the presence of NH_4_^+^ ions may minimize the negative impact of NO_3_^−^ on the Fe uptake into roots [26].In addition, it has been reported that the application of NH_4_^+^ affected the transport of Fe from old leaves and stems to young leaves resulting in a significant increase in the Fe content of young leaves [27–29]. In conclusion, providing both N forms in the correct proportion can optimize plant growth, increasing nutrient availability, and strengthen plant responses to environmental stresses [30–32].

The molecular mechanisms involved in Fe homeostasis, the signaling processes, and regulations have been investigated and summarized in annual plants e.g., Arabidopsis, tobacco, cucumber, and soybean [4, 33–36]. Studies with perennial plants often focused on applied aspects, e.g. testing of new rootstocks to prevent Fe chlorosis in apple trees [37] or citrus [38] by analyzing physiological parameters and nutrient concentrations in roots and leaves. In grapevine, up to now, only a single study analyzed the transcriptomic response of grapevine rootstocks to Fe deficiency in plants grown in hydroponic culture [11]. To cope with Fe-limiting conditions, grapevine, and other dicots plants tend to increase their absorption of Fe from the rhizosphere using a process defined as *Strategy I* (reduction strategy), which consists of (i) an increase in rhizosphere acidification through the release of H^+^, carboxylates and/or phenolic compounds to improve Fe^+ 3^ solubility, (ii) reducing chelated ferric iron (Fe^+ 3^) into ferrous iron (Fe^+ 2^) via ferric chelate reductase enzyme, and (iii) the uptake and transport of Fe^+ 2^ iron into root cells via a Fe-regulated transporter [39]. Main proteins associated to *Strategy I* Fe acquisition are involved in rhizosphere acidification by the release of protons into the rhizosphere (*AHA2*) [40], the reduction of Fe^+ 3^ chelates by ferric chelate reductase activity (*FRO2*) [41], and enter symplast pathway by *Iron-Regulated Transporter1* (*IRT1*) [42]. The transcriptional response to low Fe availability is mediated through *bHLH* (*basic helix-loop-helix*) transcription factors such as *FIT* (*FER-LIKE IRON DEFICIENCY–INDUCED TRANSCRIPTION FACTOR*) [43]. The stress response itself is regulated by phytohormones, as several studies suggest auxin and ethylene as key metabolites in Fe deficiency stress response due to their involvement in promoting the development of root hairs, besides the generation of local/long-distance signals and the regulation of root H^+^ fluxes [44, 45]. Furthermore, it has been shown that abscisic acid (ABA) is involved in both reutilization and transportation of Fe from the root to the shoot in Arabidopsis during a Fe-deficit status [46]. Conversely, cytokinin can negatively impact the expression of genes involved in Fe uptake, such as *FRO2* and *IRT1* under Fe deficiency conditions [47].

Only a few studies have attempted to investigate the effect of N-form on the response of grapevine rootstocks to Fe deficiency [2, 48, 49]. Therefore, the knowledge of this interaction, especially on the biochemical and molecular level is rather limited. Based on the available information, we hypothesize that Fe uptake into grapevine rootstocks and its translocation under Fe deficiency is mainly controlled by the genotype, while the influence of other nutrient availability is secondary. In the presented study, we used a hydroponic system to induce the Fe deficiency response in two rootstocks (Fercal: tolerant to deficiency induced Fe chlorosis; 3309Courderc: highly susceptible) and implemented the additional factor of using different N forms (nitrate and ammonium). By determining morphological, physiological, biochemical, and molecular parameters, we aim to understand genotype specific adaptation of growth and stress response focusing on Fe uptake and translocation.

## Materials and methods

### Plant material and experimental setup

The experiment was performed in 2021 in a glasshouse growth chamber under semi-controlled conditions (photoperiod:16 h of light and 8 h of darkness, light intensity of 350–500 µmol m^− 2^ s^− 1^, temperature: 22–30 °C during days and 18–25 °C during nights, relative humidity 60–70%) of the facilities of the University of Natural Resources and Life Sciences Vienna (BOKU) at the location UFT Tulln. A standardized hydroponic system (Kick-Brauckmann pot system, Stoma, Siegburg, Germany) was used to investigate three factors: the grapevine rootstock genotype [Fercal (*V.berlandieri* x *V. vinifera*) and Couderc 3309 (*V. riparia* x *V. rupestris*)], the response to Fe deficiency, and different ratios of N forms. One-year-old woody cuttings of ungrafted rootstocks with two buds were rooted in April 2021 in a mixture of perlite and peat substrate (ED73T, Einheitserde, Sinntal-Altengronau, Germany). After 4 weeks, rooted cuttings were pruned to maintain one main shoot per plant (10–15 cm) and transferred to the hydroponic pot system (5 plants per pot) filled with 7.5 L of modified half-strength Hoagland nutrient solution [50]. The treatments started 10 days after acclimation of plants to the new growth conditions. 6 mM of N were provided with different nitrate: ammonium ratio, either only as nitrate (NO_3_^−^/ NH_4_^+^ (1:0)) or as nitrate and ammonium (NO_3_^−^/ NH_4_^+^ (1:1)), both under Fe-sufficient and Fe-deficient conditions. The resulting four treatments were kept for the experimental period of 28 days: (1) N1A0/+Fe: 6 mM NO_3_^−^/ NH_4_^+^ (1:0) + 50 µM FeNa(III)-EDTA; (2) N1A0/-Fe: 6 mM NO_3_^−^/ NH_4_^+^ (1:0) + 0 µM FeNa(III)-EDTA; (3) N1A1/+Fe: 6 mM NO_3_^−^/ NH_4_^+^ (1:1) + 50 µM FeNa(III)-EDTA; (4) N1A1/-Fe: 6 mM NO_3_^−^/ NH_4_^+^ (1:1) + 0 µM FeNa(III)-EDTA.

The N was supplied as Ca(NO_3_)_2_.4H_2_O, (NH_4_) _2_SO_4_, NH_4_NO_3,_ or/and KNO_3_ with a compensation of the calcium with CaCl_2_.2H_2_O. The other elements were applied as: 1 mM MgSO_4_.7H_2_O, 0.25/0.75 mM K_2_SO_4_, 1 mM KH_2_PO_4_, 23.2 µM H_3_BO_3_, 0.31 µM CuSO_4_.H_2_O, 4.6 µM MnCl_2_.4H_2_O, 0.4 µM ZnSO_4_.H_2_O, 0.06 µM Na_2_MoO_4_.2H_2_O. The nutrient solutions were renewed once a week and evapotranspiration losses were replenished with water every second day. All pots were continuously oxygenized and the pH values of all pots were monitored daily (portable pH Meter Hi 991,300, Hanna Instruments, Woonsocket, Rhode Island, US) and adjusted to 5.7-6.0 with either 1 M NaOH or 1 M HCl.

### Plant growth and chlorosis symptoms

Standard morphometric parameters such as shoot length (Sh_L; cm), growth rate (GR; cm.day^− 1^), and specific leaf area (SLA; cm^2^.g^− 1^ dry matter) were measured at the end of the experiment from all plants (5 plants per treatment and rootstock). Fresh weight (FW) and dry weight (DW) were determined from roots, main shoot, old leaves (basal matured leaves), and young leaves (fully expanded leaves, usually the 4th to 7th leaves from the shoot tip) separately (DW after 72 h at 80 °C). The relative growth rate (RGR as g g^− 1^day^− 1^) was calculated as previously described by using the initial and final dry mass as input data [51].

**Fig. 1:**
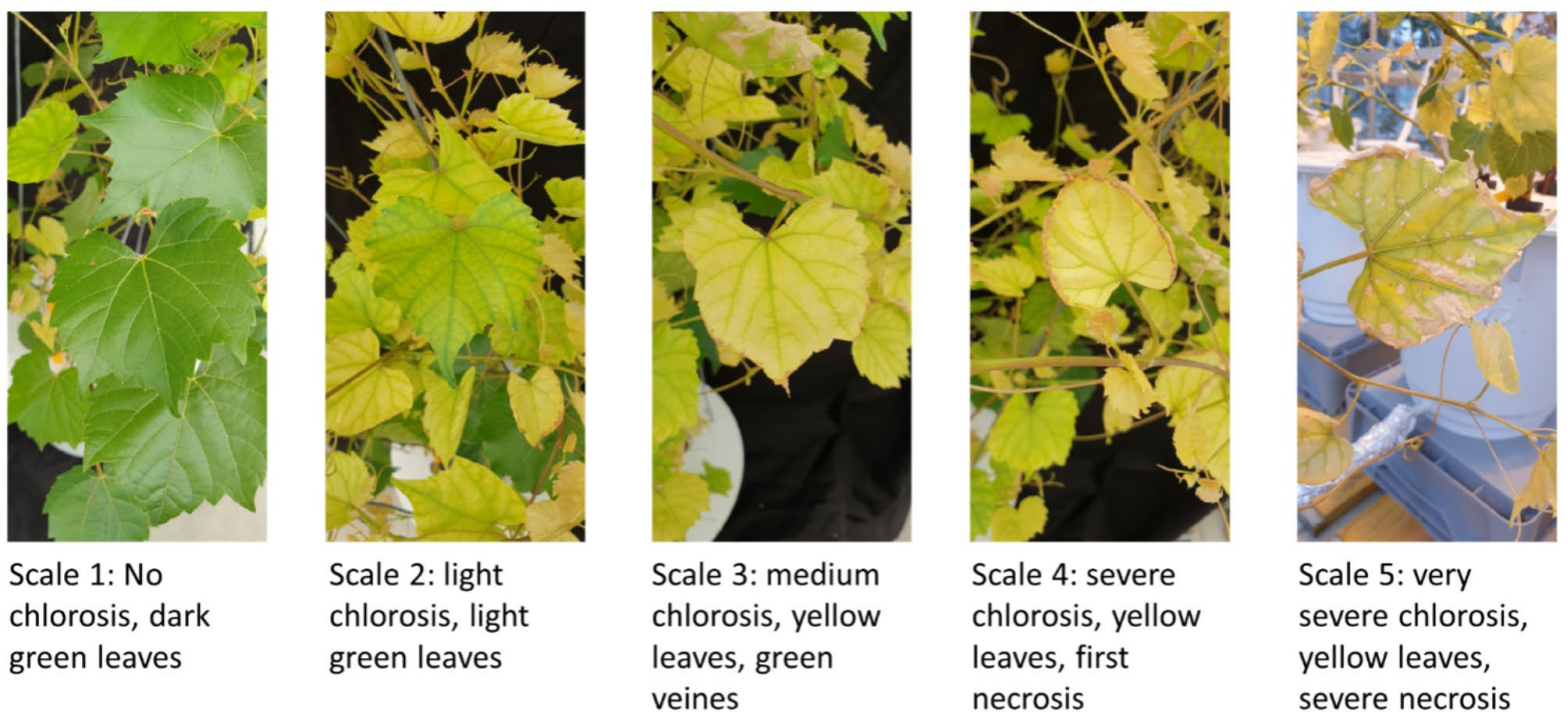
Score for Fe-deficiency chlorosis. Pictures represent the increased symptom severity with rootstock 3309 C as observed in our experiment. The chlorosis score was developed for grapevine rootstock young leaves according to the Pouget index

Chlorosis symptoms were visually evaluated on young apical leaves by applying the Pouget index [52], ranging from 0 (no symptoms, dark green leaves) to 5 (intensive chlorosis, yellow leaves seen with more than 10% necrosis) (Fig. 1).

### Chlorophyll content

During the experimental period, the chlorophyll content in young fully-expanded leaves was assessed on a weekly basis using non-destructive handheld instruments. The portable chlorophyll meter SPAD MINOLTA 502 (Konica Minolta, Inc., Osaka, Japan) gives the chlorophyll index (SPAD value), while the PolyPen RP400 UVIS (PSI Ltd., Drasov, Czech Republic) measures the leaf hyperspectral reflectance within a range from 380 to 790 nm and calculates several vegetation indices among them the NDVI (Normalized Difference Vegetation Index [53]) and the MCARI (Modified Chlorophyll Absorption in Reflectance Index [54]).

At the end of the experiment, photosynthetic pigments were extracted from fully expanded young and old leaves of plants grown under each treatment (*N* = 5), by a procedure described by [55] with modifications. Three similar leaf discs were freeze-dried for 48 h using a vacuum freeze dryer (Christ Beta 2–4 LD plus LT, Marin Christ Corporation, Osterode, Germany). 20–30 mg of freeze-dried leaf tissue were weighed in 2 mL tubes and incubated with 1.8 mL of dimethyl sulphoxide (DMSO) at 40 °C for 45 min until the tissue became colourless (Thermomixer comfort, Eppendorf, Hamburg, Germany). The tubes were then centrifuged at 13,300 rpm for 3 min at room temperature. The supernatant was transferred to a cuvette and the absorbance was read in a Spectrophotometer (Genesys, Thermo Fisher Scientific, Madison, USA) at 645, 663, and 710 nm against DMSO as blank. The concentrations of chlorophyll a, chlorophyll b, and total chlorophyll were calculated using the following equations:

chlorophyll a (mg g^− 1^) = [12.7 * (A663 - A710) − 2.69 *(A645 - A710)] * V/(1000 * W).

chlorophyll b (mg g^− 1^) = [22.9 * (A645 - A710) − 4.68 * (A663 - A710)] * V/(1000 * W).

Input data are A (absorbance of chlorophyll extract at the specific indicated wavelength), V (final volume of the solution measured), and W (mg in FW of the tissue extracted). Total chlorophyll content is summarized from chlorophyll a and b.

### Plant photosynthetic activity

Leaf transpiration rate (E, mmol H_2_O m^− 2^ s^− 1^) and stomatal conductance (*g*_*sw*_, mol H_2_O m^− 2^ s^− 1^) were measured using the porometer LI-600 (LI-COR Inc., Nebraska, USA) on a weekly basis between 10 and 14 h on clear sunny days (*N* = 5 per treatment and rootstock). Measurements were conducted at a CO_2_ concentration of 400 µmol mol^− 1^, light intensity of 10,000 µmol m^− 2^ s^− 1^, and an ambient leaf temperature and relative humidity [49].

The maximum quantum yield of the primary photochemistry (Fv/Fm) was determined using a portable hand-held instrument, the Plant Efficiency Analyzer (PEA, Hansatech Instruments Ltd., UK). Five fully-expanded young leaves were selected from each plant and were first dark adapted (∼30 min) by applying clips on each leaf and then exposed for 3 s to light at an intensity of 3500 µmol m^2^ s^− 1^. The actual photochemical efficiency of PSII (YII) was measured on the same leaves with the portable PAM-2500 chlorophyll fluorometer (Heinz Walz GmbH, Effeltrich, Germany). Pulse amplitude modulation (PAM) measurements were taken on sunny days on leaves exposed directly to solar radiation.

### Ferric eductase (FCR) enzyme activity

The FCR activity was measured according to [56]. Root tips (approximately 2 cm) were collected in 2 mL Eppendorf tubes placed on ice and transferred to the laboratory. 100 mg apical root was incubated first in 2 mL of 0.2 mM CaSO_4_ for 10 min at room temperature before transferring them to 2 mL assay solution, containing 5 mM MES-NaOH (pH 5.5), 10 mM CaSO_4_, 0.1 mM Fe(III)-EDTA, and 0.3 mM sodium Bathophenanthrolinedisulfonic acid (Na-BPDS) (all chemicals from Sigma-Aldrich, Steinheim, Germany). This step and the following incubation for 1 h were performed at room temperature in the dark. Afterward, tubes were centrifuged at 13,300 rpm for 2 min at room temperature and a 1 mL aliquot from each tube was transferred into a cuvette to measure the absorbance at 535 nm (Spectrophotometer Genesys, Thermo Fisher Scientific, Madison, USA) against BPDS as a blank. Activity of FCR is expressed as µmol g^− 1^ FW*h^− 1^ and calculated from ((A535/0.02214 µM)*(volume of assay solution (L))/((time (h)*(FW roots (g)).

### Organic acid content in roots

The organic acid contents were determined as previously reported [57]. Briefly, frozen samples of root tips were ground to powder using liquid nitrogen. 20 mg were transferred into 2 mL tubes containing 1.8 mL ddH_2_o, vortexed, and moved to an ultrasonic bath for 20 min at room temperature. Samples were placed on a shaker for 10 min at 450 rpm and then centrifuged at 13,300 rpm for 3 min. The supernatant was filtered through a 0.45 μm pore size Nylon syringe filter (Agilent Technologies, Santa Clara, United States). Finally, 600 µL of centrifuged and filtered samples were vialed and injected into the HPLC system (Dionex™ UltiMate™ 3000, Thermo Fischer Scientific). The organic acids were analyzed onto Acclaim® Organic Acid Dionex column (5 μm, 120 Å, 4.0 × 250 mm) supplied by Thermo Fisher Scientific (Ballycoolen, Dublin 15, Ireland) using the following conditions: column temperature 30 °C; injection volume 5 µL; mobile phase 0.1 M Sodium sulfate; flow rate 0.6 mL min^− 1^; and UV detection. Chromatograms were run for 40 min using a detection wavelength of 210 nm.

### Elemental analyses in plant tissues

The element concentrations of several macro (P, K, Ca, Mg, S) and micro (Fe, Mn, Zn) nutrients in grapevine root and leaves samples were determined by Inductively Coupled Plasma–Optical Emission Spectroscopy (ICP-OES 5800. Agilent Technologies. Santa Clara. USA), while the total N and C were determined by CHN analyzer (CHN IRMS Isoprime 100 Stable Isotope Ratio Mass Spectrometer, Elementar Como Italy). For ICP analyses plant samples were oven-dried for 72 h (at 60–80 °C) and ground. For each sample, 100 mg of ground powder was ashed at 550 °C in glass vials and suspended in ultrapure HNO_3_ as previously described by [21]. Element quantifications were carried out using certified multi-element standards. Regarding CHN analyses, plant leaves and roots were dried. And their total N and C contents were determined by CHN-IRMS (CHN IRMS Isoprime 100 Stable Isotope Ratio Mass Spectrometer, Elementar).

### RNA extraction, RNA seq analysis, and bioinformatics

The total RNA of root tips was extracted with the Spectrum Plant Total RNA kit (Sigma-Aldrich) from 100 mg frozen ground plant material according to the manufacturer’s protocol. The extraction was carried out in three individual samples collected from three different plants as biological replicates for each treatment. RNA quality and quantity control, mRNA library preparation, and sequencing were performed as a commercial service with Novogene Europe (Novogene, Cambridge, UK) as paired-end 150 bp sequencing on an Illumina platform NovaSeq 6000 for all 24 collected samples.

The fastq and alignment quality control utilized FastQC for raw fastq data, Qualimap for alignment assessment, and MultiQC for integrated metrics reporting [58–60], the fastq sequences were aligned to the reference genome by STAR aligner [61]. The read counting was performed by featureCounts [62] with the respective GTF/GFF file indicated below. The recent PN40024.v4 reference genome and its annotation were obtained from the INTEGRAPE website (https://integrape.eu/resources/genes-genomes/genome-accessions/). The genome annotation is available in the form of a general feature format (GFF) and a functional gene annotation was performed using the BLAST2GO [63]. 41,160 transcriptome isoforms are associated with gene ontology (GO) terms. For functional gene expression analysis, a standardized GO annotation file was transformed to the gene level. Differentially expressed genes (DEGs) were defined by a fold change of 2.0 or more and a False Discovery Rate (FDR) threshold of less than 0.05. Differential gene expression analysis and GO, as well as pathway analysis for DEGs, were performed using the integrated Differential Expression and Pathway analysis (iDEP 1.1) [64], and selected enriched biological processes were plotted using an adapted protocol as described in [65], within the ggplot2 package (v3.4.0) in R (v4.2.2; https://www.R-project.org/) and RStudio (v2023.06.1 + 524; https://www.rstudio.com/). Additionally, we conducted principal component analysis (PCA) using DESeq2 (v1.8.3) [66] and visualized it with the same ggplot2 package. The images showing gene expression related to auxins and ABA were created with BioRender.com. Genes related to the phytohormones were selected based on prior knowledge and log fold change values being used to colour-code the level of expression.

### Statistical analysis

Statistical analysis was performed using IBM SPSS statistic software (IBM Corp. Released 2020. IBM SPSS Statistics for Windows, Version 27.0. Armonk, NY: IBM Corp). Differences between groups (treatments within each rootstock and same treatment between rootstocks) were assessed using the robust Welch-ANOVA analysis of variance followed by Tukey or Games-Howell post-hoc test (α < 0.05) in case of homogeneity of variances was not ascertained (Levene-Test). Additionally, to answer some questions addressed in the discussion, a Two-Way ANOVA was calculated of selected data to understand the influence of rootstocks of one of the treatments factors in more detail. All tables were prepared using Excel 2019 (Windows 10) while figures were generated by using the SRPLOT online tool (https://www.bioinformatics.com.cn/en) and R statistical software (version 4.2.2; R Core Team 2022).

## Results

### Nitrogen-iron interaction strongly affects the phenotype of grapevine rootstocks

**Fig. 2:**
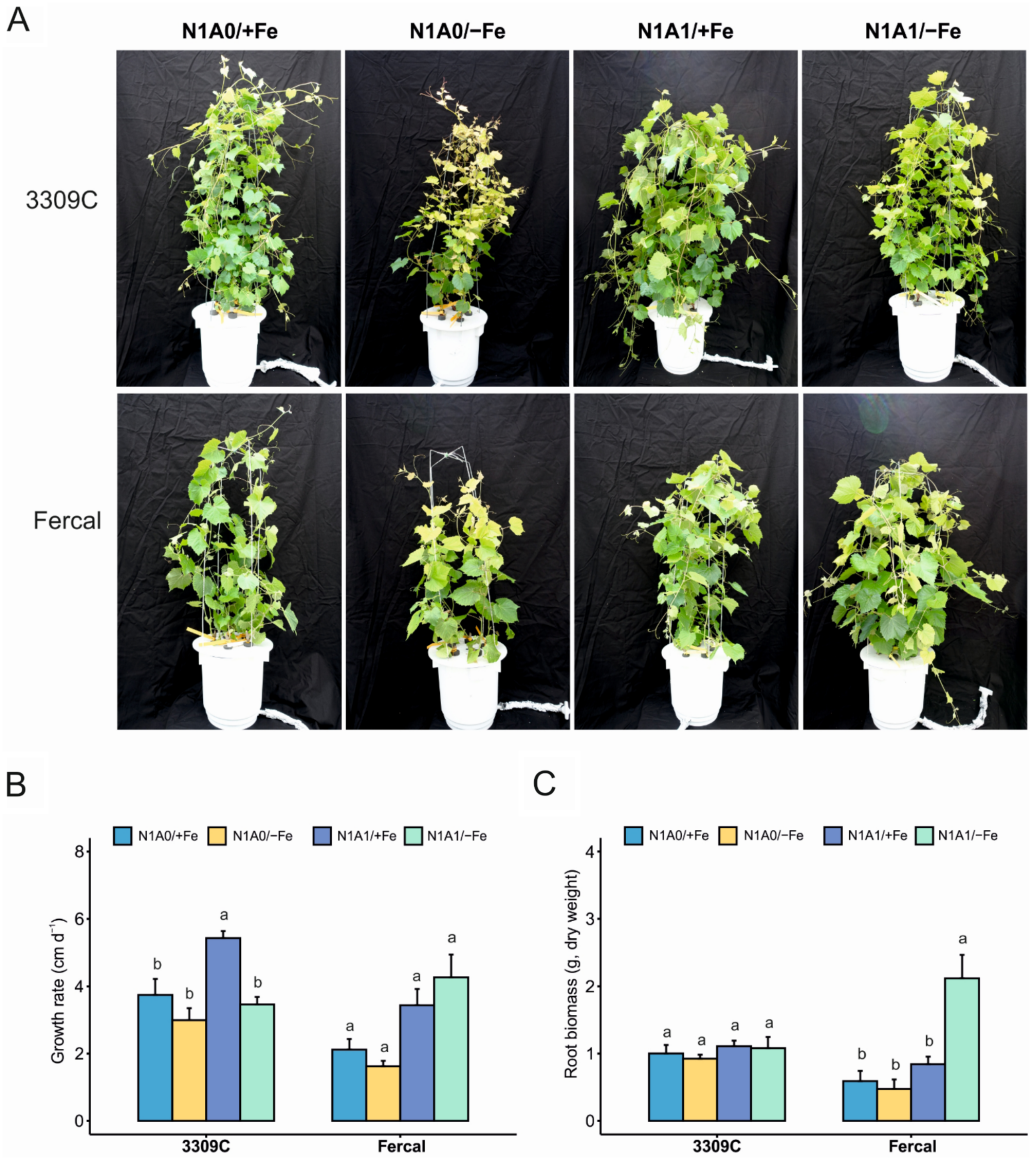
Rootstock’s phenotype and growth parameters at the end of the experiment. **A**) 3309 C and Fercal phenotype at different treatments (N1A0/+Fe; N1A0/-Fe; N1A1/+Fe; N1A1/-Fe), **B**) results of shoot growth rate (cm d^-1^), and **C**) root total biomass (g, dry weight) of both rootstocks. Values shown are means ± standard error. Significant differences between treatments for each rootstock are indicated with different letters (α < 0.05, Welch-ANOVA and Tukey or Games-Howell post hoc test, *N* = 5 per treatment and rootstock)

The rootstocks differed in developing chlorosis symptoms depending on the N source and Fe availability. Looking first at Fe-sufficient treatments (Fig. 2A; N1A0/+Fe, N1A1/+Fe), no chlorotic leaves were observed with both rootstocks. In contrast, under Fe-deficient conditions, interveinal chlorosis of young leaves appeared earlier with 3309 C from the second week of treatment with nitrate as the sole N form (N1A0/-Fe), while symptoms on leaves of Fercal were observed only after 3–4 weeks with less symptom severity (Fig. 2A, N1A0/-Fe). These symptoms were for both rootstocks, much less severe as under Fe deficiency when the nutrient solution contained both N forms (NO_3_^−^ /NH_4_^+^ (1:1); Fig. 2A, N1A1/-Fe). The strong effect of the applied N form is reflected in the treatment’s effects on the pH value of the nutrient solution (Figure S1, additional files). Minor or slightly higher pH values were determined in only nitrate nutrient solution, while the pH values were strongly decreased when both N forms were present (0.5–3.0 pH units). The influence of the rootstock on the pH value of the solution was less prominent, suggesting a similar N-form preference for N uptake.

Several growth-related parameters were differently affected in both rootstocks depending on Fe availability and available N forms (Table 1 including statistical results, Fig. 2B, C). In general, plants grown under N1A1/+Fe condition performed better as compared with N1A0/+Fe in both rootstocks. The lowest values in most growth and biomass parameters were observed with both rootstocks with treatment N1A0/-Fe. In contrast, the response to Fe deficiency when both N forms were present (N1A1/-Fe) differed between rootstocks. The growth rate with 3309 C was affected by treatments (Welch´s F(3, 8.566) = 16.744, *p* = 0.001) with enhanced values in N1A1/+Fe with a mean value of 5.42 cm d^− 1^ (Fig. 2B) resulting in the tallest plants (mean value of 152.0 cm) after 30 days of treatment (Fig. 2B; Table 1). While in rootstock Fercal, by trend the tallest plants with the highest growth rate (119.4 cm; 4.26 cm d^− 1^) were observed under Fe deficiency with both N forms (N1A1/-Fe) (Fig. 2B; Table 1), although these results have to be interpreted with care due to high data variability statistical tests were not conclusive (Welch´s F(3, 6.976) = 4.989, *p* = 0.038). The root (Welch´s F(3, 5.977) = 5.683, *p* = 0.035) and shoot (Welch´s F(3, 4.739) = 22.969, *p* = 0.003) biomass, with an increase of 151% and 67% respectively in N1A1/-Fe as compared to N1A1/+Fe (Fig. 2C; Table 1) is enhanced with rootstock Fercal, while a growth promotion translated into an increase in root or shoot biomass was not observed with rootstock 3309 C (roots: Welch´s F(3, 8.376) = 1.037, *p* = 0.425) (Fig. 2C; Table 1). In summary, both rootstocks respond slightly negative in growth with treatment N1A0/-Fe, while their response with treatment N1A1/-Fe differed substantially, with Fercal showing a growth promotion which was not observed with 3309 C.

**Table 1:** Growth and morphological parameters were determined with both rootstocks in all treatments (different nitrogen forms and Fe supply levels)

	Treatment	Shoot length	Specific leaf area (SLA)	Growth rate	Shoot	Leaves	Root	Relative growth rate	Root/shoot ratio
		(cm)	(cm^2^ g^− 1^ DM)	(cm d^− 1^)	dry weight	dry weight	dry weight	(g g^− 1^ d^− 1^)	
					(g)	(g)	(g)		
3309 C	N1A0 / +Fe	104.8 ± 29.8 b	395.85 ± 90.3 ab	3.74 ± 1.1 b	8.93 ± 3.2 a	2.95 ± 0.6 ab	1.00 ± 0.3 a	0.027 ± 0.01 ab	0.090 ± 0.03 a
	N1A0 / -Fe	83.8 ± 22.3 b	345.51 ± 120.8 ab	2.99 ± 0.8 b	7.45 ± 2.2 a	1.07 ± 0.3 b	0.92 ± 0.1 a	0.020 ± 0.01 b	0.111 ± 0.03 a
	N1A1 / +Fe	152.0 ± 13.1 a	408.21 ± 46.1 b	5.42 ± 0.5 a	10.92 ± 1.8 a	3.83 ± 1.0 a	1.11 ± 0.2 a	0.034 ± 0.01 a	0.074 ± 0.01 a
	N1A1 / -Fe	96.9 ± 13.8 b	347.69 ± 64.6 a	3.46 ± 0.5 b	8.13 ± 1.6 a	2.45 ± 0.9 ab	1.08 ± 0.4 a	0.026 ± 0.01 ab	0.099 ± 0.02 a
	Welch-ANOVA	F(3, 8.582) = 16.670, *p* = 0.001	* F(3, 40.119) = 4.570, *p* = 0.008	F(3, 8.566) = 16.744, *p* = 0.001	F(3, 8.701) = 2.671, *p* = 0.113	F(3, 7.474) = 12.019, *p* = 0.003	F(3, 8.376) = 1.037, *p* = 0.425	F(3, 8.736) = 3.607, *p* = 0.060	F(3, 7.704) = 4.183, *p* = 0.049
Fercal	N1A0 / +Fe	59.3 ± 18.0 a	262.91 ± 61.4 b	2.12 ± 0.6 a	9.33 ± 2.5 ab	1.43 ± 0.6 b	0.59 ± 0.3 b	0.013 ± 0.01 ab	0.070 ± 0.01 a
	N1A0 / -Fe	45.5 ± 9.0 a	319.93 ± 58.3 ab	1.63 ± 0.3 a	7.09 ± 0.9 b	1.55 ± 0.1 b	0.48 ± 0.3 b	0.005 ± 0.00 b	0.082 ± 0.04 a
	N1A1 / +Fe	96.1 ± 30.0 a	368.68 ± 115.1 a	3.43 ± 1.1 a	7.95 ± 2.3 b	2.70 ± 1.0 ab	0.84 ± 0.2 b	0.012 ± 0.01 ab	0.075 ± 0.01 a
	N1A1 / -Fe	119.4 ± 42.0 a	385.21 ± 98.6 a	4.26 ± 1.5 a	13.28 ± 0.9 a	4.65 ± 1.0 a	2.12 ± 0.6 a	0.024 ± 0.00 a	0.113 ± 0.02 a
	Welch-ANOVA	* F(3, 7.025) = 5.227, *p* = 0.033	F(3, 29.856) = 6.564, *p* = 0.002	* F(3, 6.976) = 4.989, *p* = 0.037	F(3, 4.739) = 22.969, *p* = 0.003	F(3, 3.977) = 7.461, *p* = 0.041	F(3, 5.977) = 5.683, *p* = 0.035	* F(3, 5.017) = 33.376, *p* = 0.001	F(3, 5.294) = 3.065, *p* = 0.124

Presented values are mean values with standard error. Significant differences between treatments for each rootstock are indicated with different letters. (α < 0.05, Welch-ANOVA and Tukey or Games-Howell post hoc test, *N* = 5 per treatment and rootstock)

* significant LeveneTest

### Plant physiology parameters describe chlorosis severity induced by treatments

**Fig. 3:**
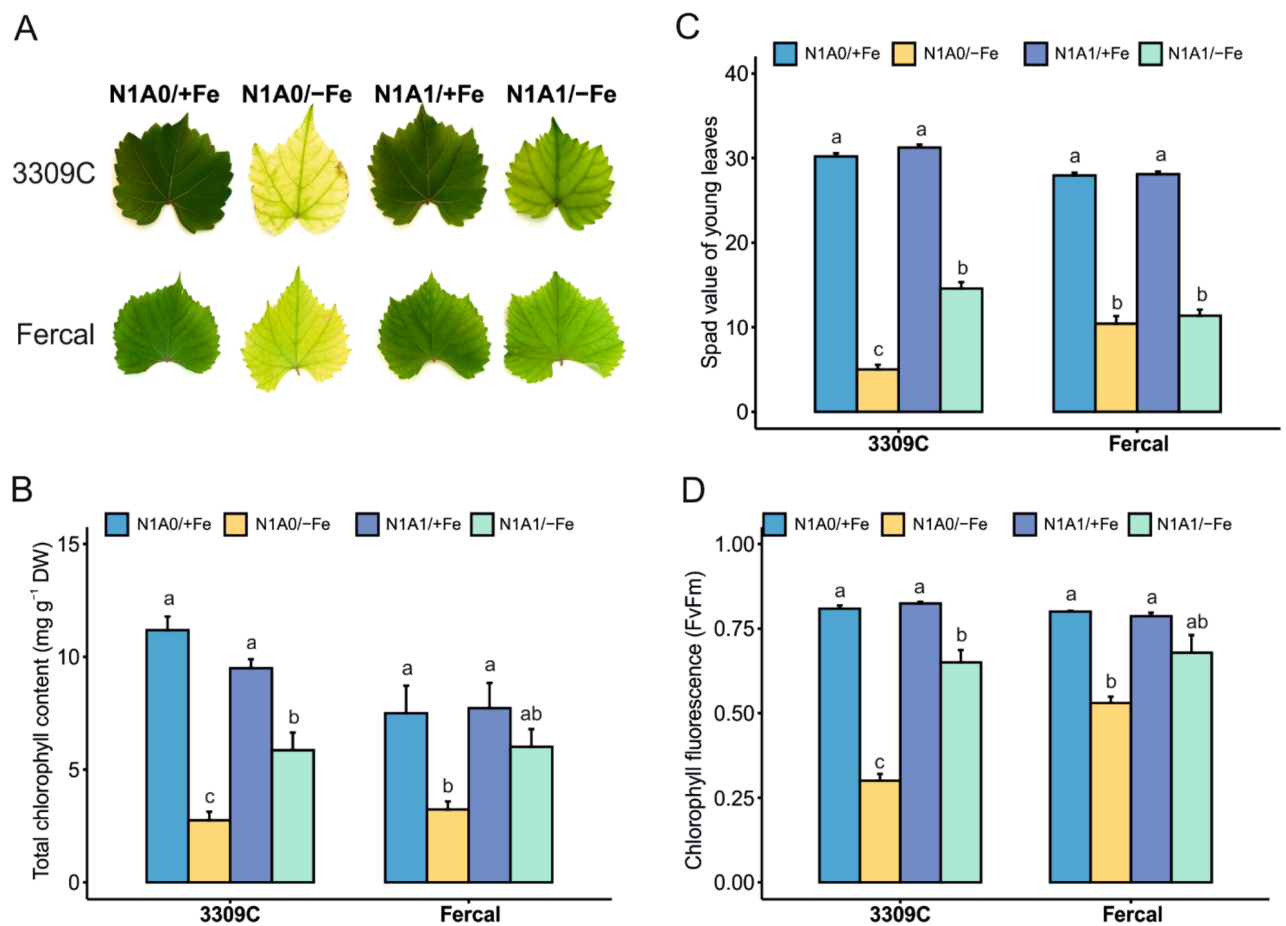
Direct and indirect evaluation of leaves chlorophyll content and chlorophyll fluorescence. (**A**) Iron deficiency chlorosis visual symptoms with both rootstocks and all treatments (N1A0/+Fe; N1A0/-Fe; N1A1/+Fe; N1A1/-Fe), (**B**) total chlorophyll concentration in young leaves, (**C**) non-destructive assessment of chlorophyll content by measuring the SPAD index, and (**D**) maximum photochemical rate of photosynthesis (FvFm). Values shown are means ± standard error and significant differences between treatments for each rootstock are indicated with different letters. (α < 0.05, Welch-ANOVA and Tukey or Games-Howell post hoc test, *N* = 5 per treatment and rootstock)

Chlorosis symptom severity was evaluated by measuring the chlorophyll content in young leaves and via indirect non-destructive measurements with hand-held instruments. The total chlorophyll content was affected by the treatments in both rootstocks (*N* = 5; Fercal: Welch´s F(3, 7.028) = 8.426, *p* = 0.010; 3309 C: Welch´s F(3, 8.637) = 61.494, *p* < 0.001) (Fig. 3A, B), especially under Fe deficient conditions, while in both rootstocks no differences were observed with different N forms under Fe-sufficient conditions. Differences appeared under Fe-limiting conditions with graver symptoms and reduced total chlorophyll content, especially when N was available only as nitrate (N1A0/-Fe) (Fig. 3A, B). Non-destructive measurements of chlorophyll content with the SPAD meter, relate to the determined total chlorophyll content (Fig. 3C), especially with rootstock 3309 C, while with Fercal the values obtained with N1A1/-Fe would overestimate the stress severity. In both rootstocks observed differences (*N* = 5; Fercal: Welch´s F(3, 16.780) = 247.525, *p* < 0.001; 3309 C: Welch´s F(3, 17.304) = 645.625, *p* < 0.001) were related to the reduced values obtained under Fe-deficiency (Fig. 3C). The photosynthesis of plants was affected by the treatments (*N* = 5; Fercal: Welch´s F(3, 3.467) = 48.769, *p* = 0.002; 3309 C: Welch´s F(3, 3.875) = 165.291, *p* < 0.001) with reduced values for the maximum potential quantum efficiency of photosystem II (Fv/Fm) of dark-adapted leaves (Fig. 3D) or similarly for the actual photochemical efficiency (YII) of light-exposed leaves (Table 2). Both parameters were significantly reduced in Fe-deficient treatments with both N forms with the lowest values observed for rootstock 3309 C, thereby reflecting nicely the stress severity. In contrast, the stomatal conductance (g_sw_) and transpiration rate (E) of the measured young leaves did not change significantly in response to Fe availability or N forms in 3309 C, while some variation was detected for Fercal, especially by trend higher values of g_sw_ and E were observed when both N forms were present. (Table 2).

**Table 2:** Effect of iron availability and nitrogen forms treatments on stomatal conductance (g_sw_), transpiration rate (E), and efficiency of photosynthesis (YII) of different grapevine rootstocks

Genotype	Treatment	Stomatal Conductance	Transpiration Rate	Efficiency of photosynthesis (Y(II)) young leaves
		(mol m^− 2^ s^− 1^)	(mmol m^− 2^ s^− 1^)	
3309 C	N1A0 / +Fe	0.24 ± 0.04 a	4.11 ± 1.53 a	0.68 ± 0.03 a
	N1A0 / -Fe	0.37 ± 0.07 a	4.31 ± 0.61 a	0.21 ± 0.04 c
	N1A1 / +Fe	0.36 ± 0.13 a	5.39 ± 1.41 a	0.66 ± 0.03 a
	N1A1 / -Fe	0.42 ± 0.18 a	6.09 ± 1.72 a	0.53 ± 0.06 a
	Welch-ANOVA	F(3, 10.793) = 1.477, *p* = 0.276	F(3, 10.070) = 3.214, *p* = 0.070	* F(3, 5.334) = 96.979, *p* < 0.001
Fercal	N1A0 / +Fe	0.23 ± 0.04 ab	3.03 ± 1.35 ab	0.67 ± 0.01 ab
	N1A0 / -Fe	0.14 ± 0.06 b	2.78 ± 0.91 b	0.31 ± 0.09 b
	N1A1 / +Fe	0.35 ± 0.14 a	4.32 ± 1.36 a	0.65 ± 0.02 a
	N1A1 / -Fe	0.32 ± 0.05 a	4.12 ± 1.09 ab	0.54 ± 0.07 ab
	Welch-ANOVA	* F(3, 16.257) = 17.830, *p* < 0.001	F(3, 17.387) = 4.121, *p* = 0.022	* F(3, 3.587) = 13.490, *p* = 0.020

Presented values are mean values with standard error. Significant differences between treatments for each rootstock are indicated with different letters. (α < 0.05, Welch-ANOVA and Tukey or Games-Howell post hoc test, *N* = 5 per treatment and rootstock)

* significant Levene Test

### Root biochemical adaptation for nutrient uptake

**Fig. 4:**
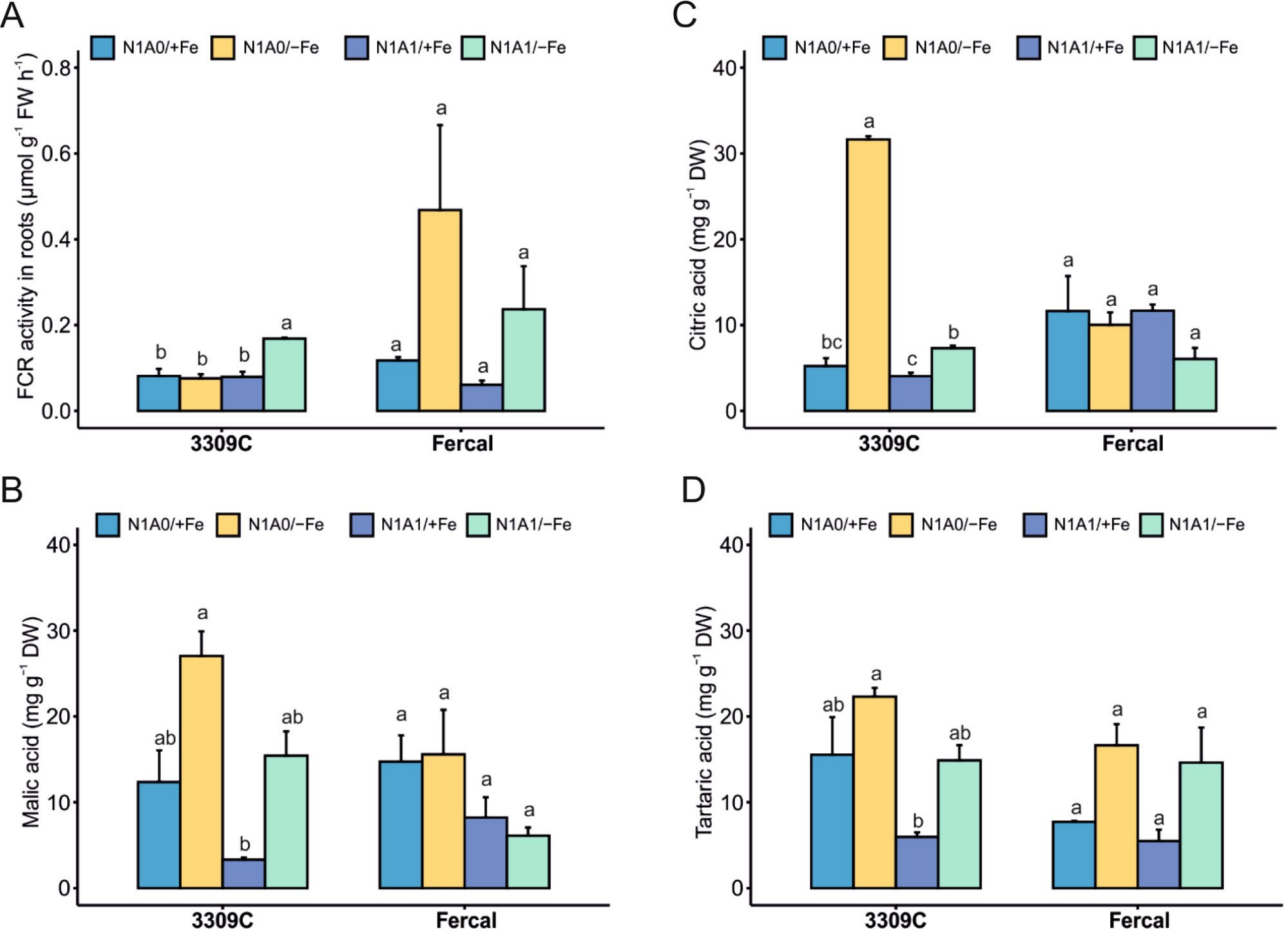
Root tip biochemistry results. (**A**) Ferric chelate reductase activity in root tips, (**B**) concentration of malic acid in root tips, (**C**) concentration of citric acid, and, (**D**) concentration of tartaric acid in the root tips of plants under all treatments (N1A0/+Fe; N1A0/-Fe; N1A1/+Fe; N1A1/-Fe). Results were obtained after 28 days of treatment application with both grapevine rootstocks. Values shown are means ± standard error and significant differences between treatments for each rootstock are indicated with different letters. (α < 0.05, Welch-ANOVA and Tukey or Games-Howell post hoc test, *N* = 3 per treatment and rootstock)

The ferric chelate reductase (FCR) activity is an essential enzyme for Fe uptake and our results showed some interesting trends differing with Fercal and 3309 C, although high variability of data makes general conclusions difficult (*N* = 3; Fercal: Welch´s F(3, 3.969) = 6.351, *p* = 0.054; 3309 C: Welch´s F(3, 3.557) = 37.342, *p* = 0.004). By trend, FCR activity was enhanced in Fercal under Fe deficiency (Fig. 4A) with both N treatments, an observation which needs further validation. On the contrary, in rootstock 3309 C there was no increase in FCR activity in plants grown under Fe deficiency when only nitrate was applied (N1A0), while when both N sources were available a slightly higher activity was measured (Fig. 4A).

The organic acids’ content was quantified in root tips to evaluate their potential release as root exudates. In general, the measured quantities of malic and tartaric acid were higher in root tips compared to citric and oxalic acids and organic acids were enhanced in 3309 C while with Fercal a similar, but not significant trend was observed (Fig. 4B-D) (Malic acid: *N* = 3; Fercal: Welch´s F(3, 3.811) = 2.511, *p* = 0.203; 3309 C: Welch´s F(3, 3.382) = 21.763, *p* = 0.011; citric acid: *N* = 3; Fercal: Welch´s F(3, 4.054) = 3.720, *p* = 0.117; 3309 C: Welch´s F(3, 4.295) = 875.649, *p* < 0.001; tartaric acid: *N* = 3; Fercal: Welch´s F(3, 3.364) = 4.462, *p* = 0.110; 3309 C: Welch´s F(3, 3.869) = 52.635, *p* = 0.001).

The values obtained from 3309 C were highly increased under Fe deficiency (Fig. 4B-D), with both N treatments, but higher absolute values were determined when nitrate was the only available N source.

In summary, we observed a boost of some organic acid contents in root tips with Fe-deficient treatments especially with 3309 C rootstock, while in Fercal enhance but not significant different values between treatments were recorded. On the other hand, FCR activity seems to be induced in Fercal root tips, while the enzyme activity was only enhanced when both N forms were present in 3309 C.

### Effects of treatments on nutrient content profiles in different plant tissues

**Fig. 5:**
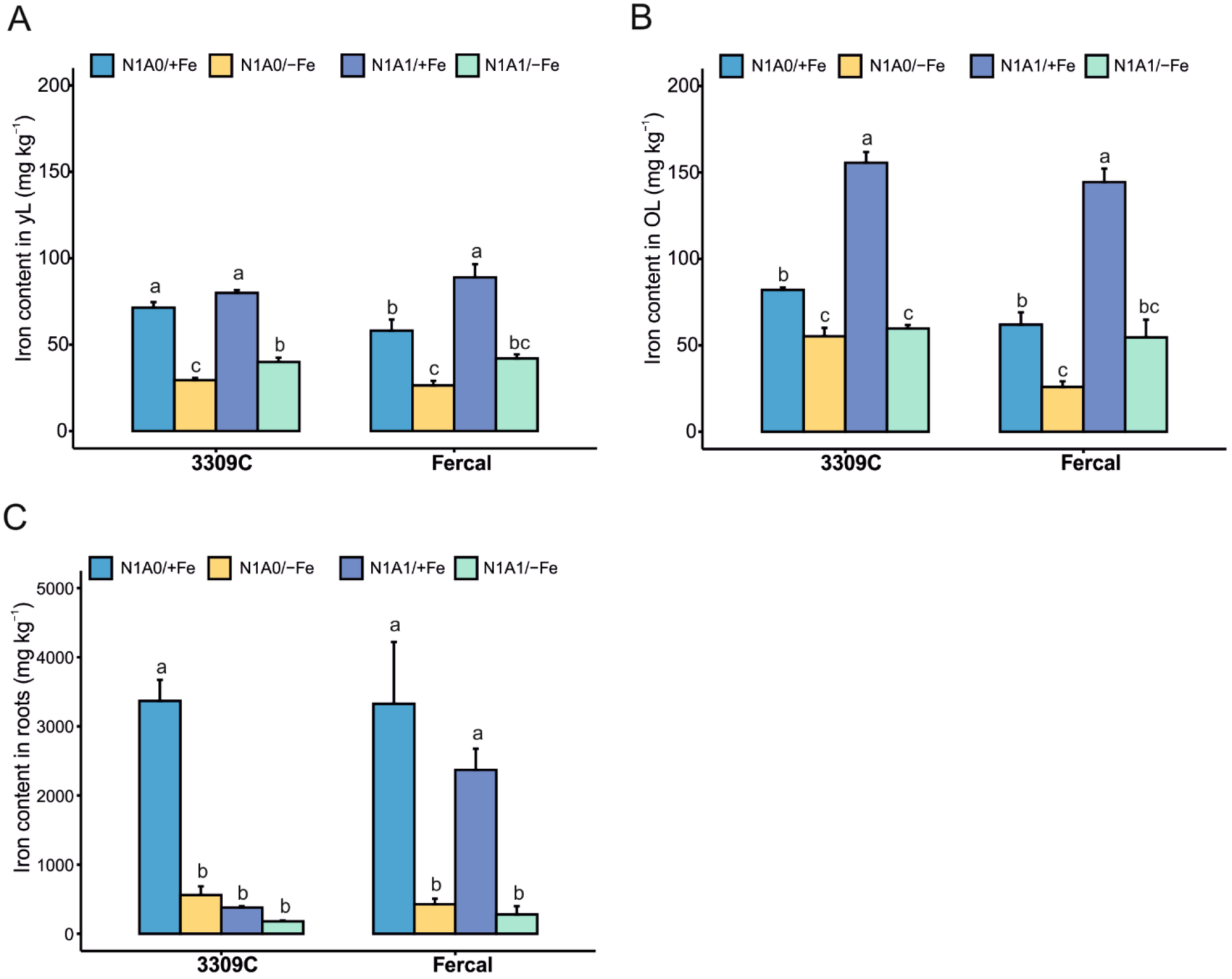
Plant iron contents. (**A**) Iron content of young leaves (yL), (**B**) old leaves (OL), and (**C**) in roots of the two grapevine rootstocks under the treatments: N1A0/+Fe; N1A0/-Fe; N1A1/+Fe; N1A1/-Fe. Results were obtained after 28 days of treatment application with both grapevine rootstocks. Values shown are means ± standard error. Significant differences between treatments for each rootstock are indicated with different letters (α < 0.05, Welch ANOVA and Tukey or Games-Howell post hoc test, *N* = 5 per treatment and rootstock)

The analysis of nutrients content’ in different tissues showed a dynamic Fe concentration influenced by the rootstock genotype and the available N forms under Fe-deficient conditions (Fig. 5A-C). In general, the Fe concentration in roots was much higher compared to old and young leaves, when Fe was supplied in the nutrient solution, and substantially dropped under Fe deficiency. In roots, the Fe content was affected by treatments in both rootstocks (*N* = 5; Fercal: Welch´s F(3, 7.088) = 32.108, *p* < 0.001; 3309 C: Welch´s F(3, 7.608) = 55.532, *p* < 0.001). High amounts of Fe were accumulated under Fe-sufficient conditions with N1A0 treatment in 3309 C and Fercal (Fig. 5C). High amounts were also observed with Fercal with N1A1/+Fe treatment, while this accumulation was not observed with 3309 C (Fig. 5C). In old leaves the Fe content was significantly affected by treatments in both rootstocks (*N* = 5; Fercal: Welch´s F(3, 4.002) = 51.396, *p* = 0.001; 3309 C: Welch´s F(3, 4.039) = 65.436, *p* = 0.001). Fe content was enhanced when both N forms were present (N1A1/+Fe) in both rootstocks, while in young leaves this effect was only observed with Fercal (Fig. 5A, B). The determined Fe contents in leaves under control conditions were very similar. The Fe deficiency effect was comparable for both rootstocks in leaves, although the absolute numbers in reduction differed slightly (Table S1). The Fe concentration in chlorotic leaves was 29.4 mg kg^− 1^ DW and 26.4 mg kg^− 1^ DW for 3309 C and Fercal, in control leaves were 71.4 mg kg^− 1^ DW and 58.1 mg kg^− 1^ DW for 3309 C and Fercal, respectively.

Apart from Fe, a summary of all analyzed nutrients is presented in the additional files (Table S1), as well as the correlation of the leaf chlorophyll content with Fe content and the hyperspectral leaf indices (NDVI, MCARI; see Figure S2). Minor changes were observed in N levels in the different tissues of both rootstocks, specifically Fercal, while the N concentration was only affected by the treatment in old leaves of 3309 C. In roots, the N content with 3309 C was not affected by treatments, while in Fercal highest N concentration was found in N1A1/+Fe.

In terms of non-destructive assessment of total leaf chlorophyll content, the hyperspectral leaf reflectance indices NDVI and MCARI were similar and suitable to give an estimation for both rootstocks with correlation coefficients of 0.66 and 0.61 respectively (Figure S2 B, C). On the contrary, the correlation between total chlorophyll content and Fe content in young leaves was affected by the rootstock genotype (Figure S2 A; R2 3309 C = 0.73; R2 Fercal = 0.51).

Significant changes in the other macronutrient and micronutrient contents reflect changes in the pH values of the nutrient solution. In general, the concentration of Mn in young leaves and roots of both rootstocks was higher under Fe deficiency, while higher concentrations of Mn were observed in roots when N was supplied as nitrate. Interestingly, we observed by trend higher concentrations of K in Fercal leaves, although this results were not significantly affected by the treatment, it could hint towards the role of potassium in alleviating Fe deficiency stress through reutilization of Fe from the root and promoting Fe transportation towards leaves as proposed [6, 67].

### Transcriptomic profiles separate grapevine rootstocks specifically under Fe deficiency

**Fig. 6:**
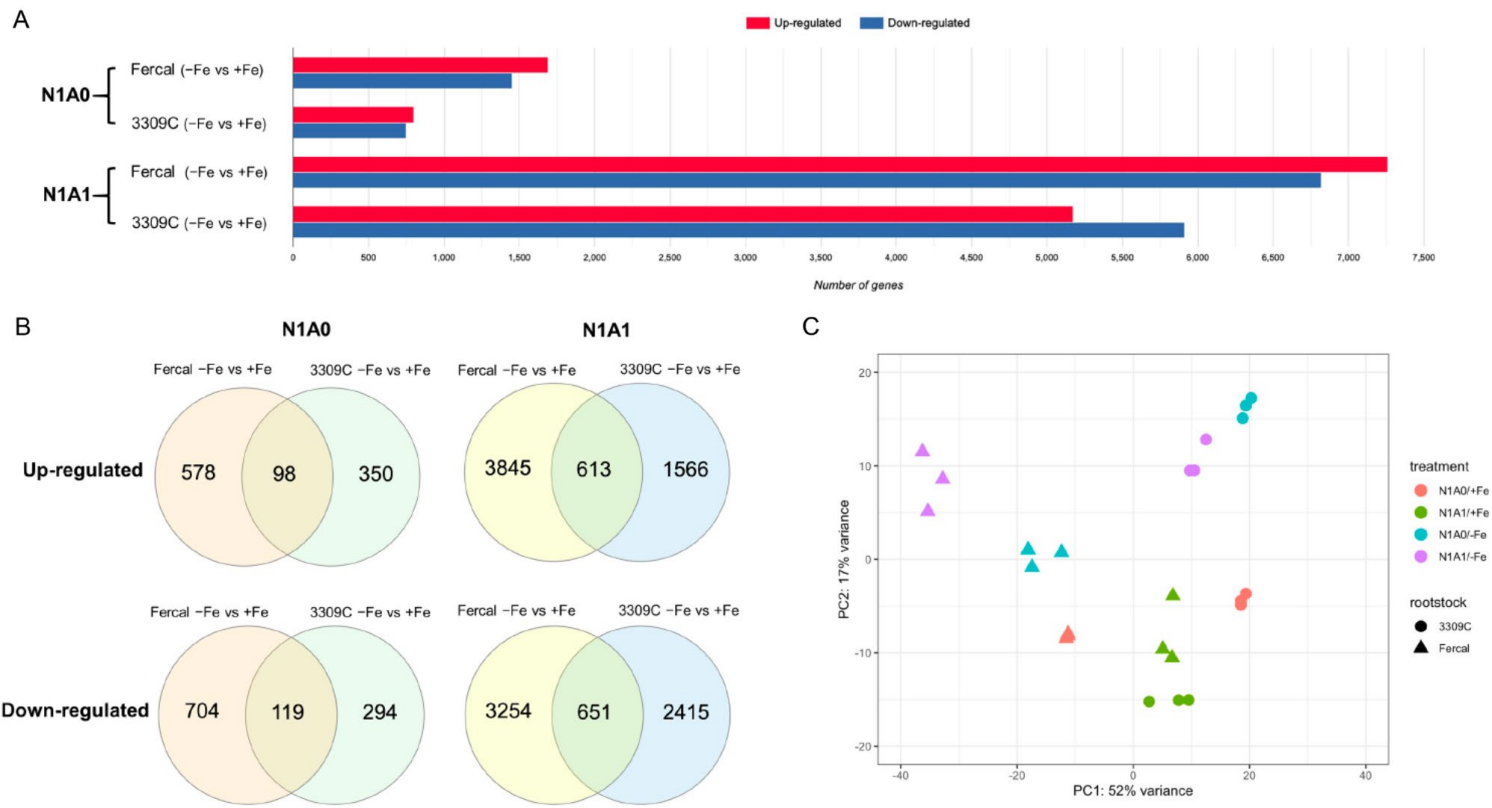
Differentially expressed genes. (**A**) Bar graph of up-and down-regulated genes in each comparison, (**B**) Venn diagram showing common and unique DEGs (|log_2_ FC | ≥ 2, FDR < 0.05) in both genotypes, and (**C**) PCA (Principal Component Analysis) plot with the top-most variable genes generated from DeSeq2 showing variation between genotypes and treatments. Genotypes are differentiated by different shapes: 3309 C (circles), and Fercal (triangles), and treatments are differentiated by different colors

Principal Components Analysis (PCA) confirmed a close similarity of transcriptional profiles of biological replicates within each treatment (Fig. 6C) and determined a clear separation between rootstocks and treatments on the basis of their transcriptional profiles. Rootstock genotypes were separated along the first component axis (PCA1), while Fe deficiency treatments tended to be clustered along the second component axis (PCA2).

In general, the numbers of differentially expressed genes (DEGs) between comparisons were rather high, ranging from 1551 to 14,078 (Fig. 6A). Thereby, follow-up analysis was performed with a cut-off of | log_2_ (Fold Change) | ≥ 2 and FDR < 0.05 for both rootstocks and all treatment comparisons (Fig. 6B). In both rootstocks, the number of DEGs caused by Fe deficiency was lower when only nitrate was available as N source (861 and 1449 in 3309 C and Fercal, respectively) than when equal amounts of nitrate and ammonium were available in the nutrient solution (5245 and 8363 in 3309 C and Fercal, respectively). Hence, regardless of N forms, there was a higher number of DEGs caused by Fe deficiency in Fercal than in 3309 C roots.

**Fig. 7:**
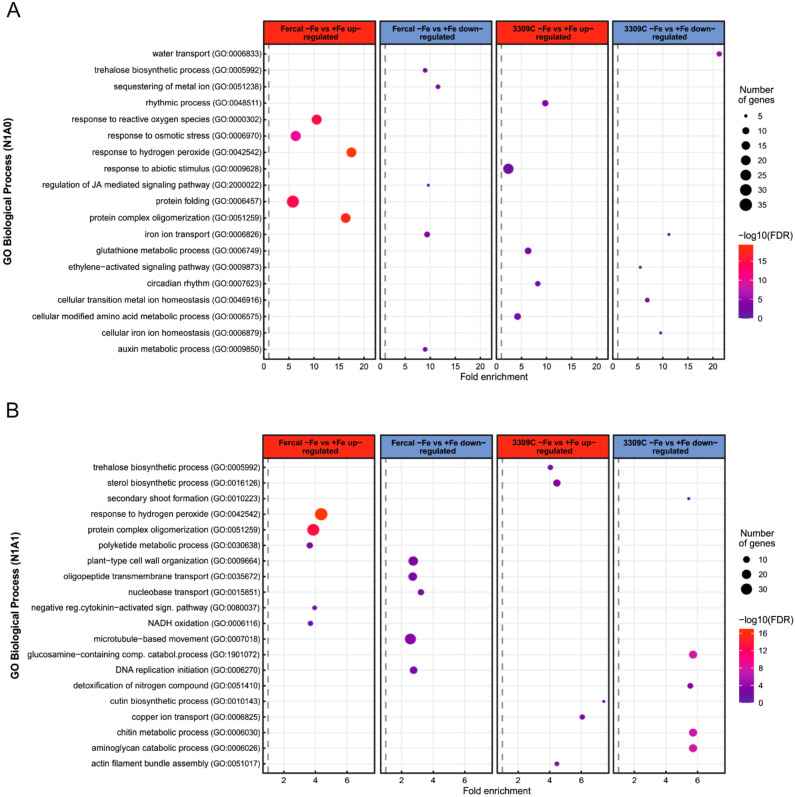
Gene Ontology pathway enrichment analysis of differently expressed genes. (**A**) The top 5 enriched GO terms of biological process related to up-regulated and down-regulated DEGs in Fercal (-Fe vs. + Fe), and 3309 C (-Fe vs. + Fe) under N1A0 form. (**B**) The top 5 enriched GO terms of biological process related to up-regulated and down-regulated DEGs in Fercal (-Fe vs. + Fe), and 3309 C (-Fe vs. + Fe) under N1A1 form. The size of the dots represents the number of genes in each pathway and the color of the dots represents the -log_10_ of False Discovery Rate (FDR)

The Gene Ontology (GO) enrichment analysis of DEGs helped us to identify key biological processes (BP) and molecular functions (MF) affected by Fe deficiency in the two rootstock genotypes and with the different N treatments in the nutrient solution: only nitrate or both nitrate and ammonium forms of N. In order to extract the most relevant information, the top gene-enriched GO terms (down-regulated and up-regulated by Fe deficiency) were identified for each treatment (N1A0, N1A1), and for each rootstock separately (Table S2, S3, S4, S5) and only 5 selected up-regulated as well as down-regulated pathways belonging to the biological processes are presented in (Fig. 7). Under N1A0 treatment in both genotypes, Fe deficiency induced the expression of genes involved in iron ion binding (MF/GO:0005506, 27 in Fercal and 30 in 3309 C), although in parallel suppressed the expression of genes involved in iron ion transport (BP/ GO:0006826, 8 in Fercal, and 5 in 3309 C). The majority of uniquely differentially up-regulated genes in Fercal (-Fe vs. + Fe) were significantly enriched for stress responses such as response to hydrogen peroxide (BP/GO:0042542, 22 genes), response to reactive oxygen species (BP/GO:0000302, 23 genes), and response to osmotic stress (BP/ GO:0006970, 24 genes). While the unique up-regulated genes in 3309 C (-Fe vs. + Fe) were significantly enriched for genes involved in the glutathione metabolic process (BP/ GO:0006749, 10 genes) and response to abiotic stimulus (BP/ GO:0009628, 24 genes).

Among the down-regulated genes in both rootstocks, some were associated with phytohormones. Fercal (-Fe vs. + Fe) showed a down-regulation of 6 genes related to the auxin metabolic process (BP/ GO:0009850), whereas 3309 C (-Fe vs. + Fe) showed a down-regulation of 5 genes related to the ethylene-activated signalling pathway (BP/ GO:0009873) (Fig. 7A).

In Fercal, under N1A1 treatment, few biological processes related to up-regulated DEGs (-Fe vs. + Fe) overlapped with GO terms under N1A0 treatment, e.g., stress response involving hydrogen peroxide (BP/ GO:0042542, 39 genes). In addition to stress responses in Fercal, the down-regulation of the cytokinin-activated signalling pathway (BP/ GO:0080037, 6 genes) was enriched. In 3309 C, several differentially expressed genes involved in different biological processes and molecular functions such as copper ion transport (BP/ GO:0006825, 7 genes), trehalose biosynthetic process (BP/GO:0005992, 7 genes), and sucrose synthase activity (MF/ GO:0016157, 5 genes) were enriched. (Fig. 7B; Table S3).

Additionally, cell wall modifying enzymes, such as xyloglucan:xyloglucosyl transferase (MF: GO:0016762) were enriched in both rootstocks, whereby Fercal had a higher number of up-regulated genes involved in this pathway (21 genes) as compared to 3309 C (11 genes) (Table S3).

### Nitrogen forms and Fe deficiency influence phytohormones signaling

**Fig. 8:**
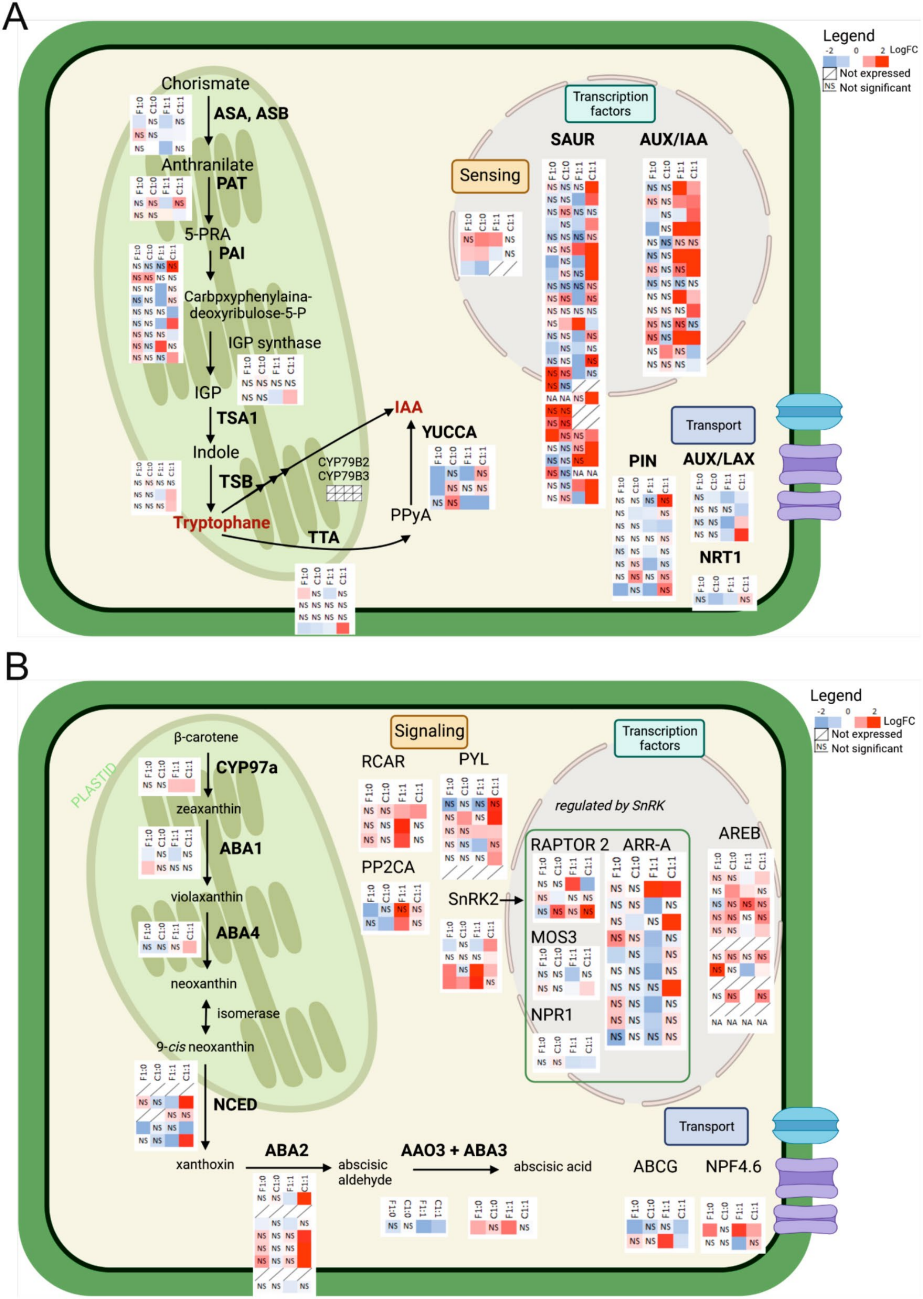
Phytohormones pathways. DEGs involved in the auxin and ABA signaling pathways in both grapevine rootstocks. (**A**) Auxin synthesis, transport, sensing, and transcription factors pathway, and (**B**) Abscisic acid synthesis, transport, signaling, and transcription factors pathway. The absolute values of log_2_FC ≥ 2 and FDR < 0.05 were used as thresholds to identify statistically significant DEGs. Color of the box indicates up (red) and down (blue)-regulated genes. / - not expressed, NS - not statistically significant, F1:0 - Fercal N1A0 (-Fe vs. + Fe), C1:0–3309 C N1A0 (-Fe vs. + Fe), F1:1 - Fercal N1A1 (-Fe vs. + Fe), C1:1–3309 C N1A1 (-Fe vs. + Fe). The detailed DEGs are listed in supplementary Table S6

In accordance with the published literature, in which auxin and abscisic acid have been shown to be among the primary phytohormones that regulate the responses of plants to Fe deficiency [68, 69]., also our transcriptomic data showed that several genes differentially regulated by Fe deficiency are involved in the biosynthesis and signaling of plant phytohormones. We plotted 80 and 70 of them on the putative auxin and ABA metabolic pathways, respectively (Fig. 8A, B).

In the auxin metabolic pathway six genes for Aux/IAA family transcription factors, (*Vitvi09g00437*, *Vitvi05g00838*, *Vitvi09g00436*, *Vitvi05g00271*, *Vitvi07g00687*, and *Vitvi14g00483*) were highly up-regulated by Fe deficiency in both rootstocks and additional three genes only in 3309 C (*Vitvi07g00042*, *Vitvi05g00630*, and *Vitvi09g00336*) when supplied with both N forms, while they were not up-regulated when supplied only with nitrate. In 3309 C additional 12 (*Vitvi03g04099*, *Vitvi18g01093*, *Vitvi18g01091*, *Vitvi09g01982*, *Vitvi09g01984*, *Vitvi03g01363*, *Vitvi04g02075*, *Vitvi03g01359*, *Vitvi03g01367*, *Vitvi01g01714*, *Vitvi04g01831* and *Vitvi11g00033*) genes for SAUR family transcription factors were highly up-regulated by Fe deficiency when supplied with both N forms, while they were not up-regulated when supplied only with nitrate. Among them, only two genes (*Vitvi04g01831* and *Vitvi11g00033*) were significantly up-regulated by Fe deficiency also in Fercal.

when supplied with both N forms. Besides, some up-regulated genes by Fe deficiency were found also in the auxin synthesis pathway. Also, these genes were up-regulated only when supplied with both N forms.

Similarly in ABA signaling pathways, most of the 15 genes belong to (RCAR, PYL, PP2C, and SnRK2) families were highly up-regulated only when both forms of N were provided. Of these, three genes (*Vitvi15g00997*, *Vitvi08g00768*, and *Vitvi18g00440*) were highly up-regulated in both rootstocks, while four genes only in Fercal (*Vitvi02g00119*, *Vitvi16g01226*, *Vitvi13g00344*, and *Vitvi07g01323*), and other four genes (*Vitvi13g00114*, *Vitvi02g00695*, *Vitvi07g02005* and *Vitvi12g01972*) only in 3309 C. Also, in the ABA synthesis pathway and transcription factors involved in ABA metabolism, the majority of up-regulated genes belonged to 3309 C supplied with both N forms.

Based on the results above, Fe deficiency seems to lead to an increase in the expression of several genes involved in auxin and ABA signaling pathways in the roots of both rootstocks under N1A1/-Fe treatment, which supports the suggestions made already regarding the role of these phytohormones in the regulation of the Fe deficiency responses in *strategy I* plants. However, more studies are required to clarify the role of N form in the activation of these phytohormones signaling pathways under Fe deficiency in grapevine rootstocks.

### Iron uptake and translocation genes respond mainly to the different fe availability

**Fig. 9:**
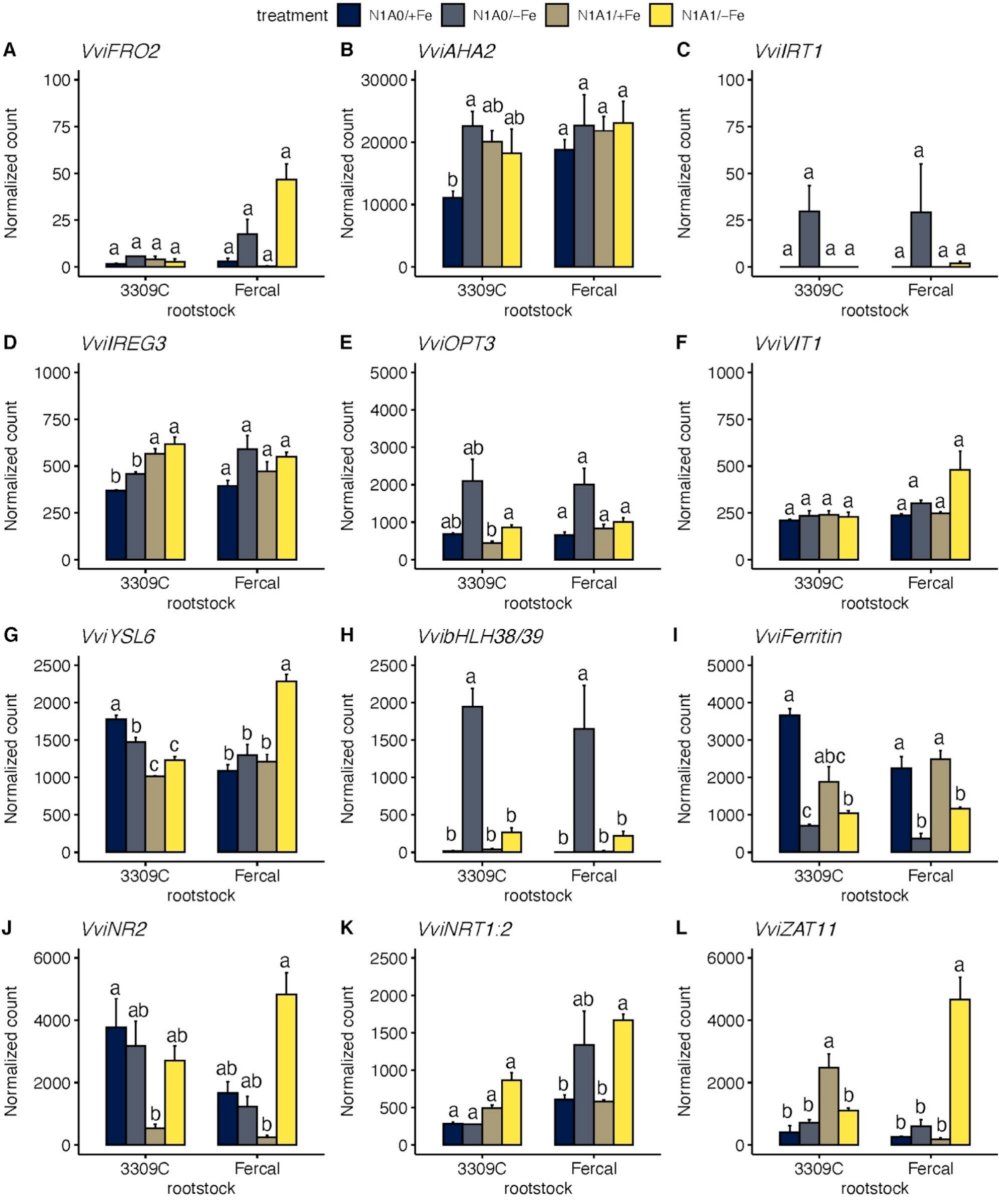
Expression of genes involved in Fe uptake and translocation mechanisms. Expression of genes, as normalized counts, involved in Fe uptake and transport in both rootstocks and all tested treatments. Only nitrate as nitrogen source (N1A0); 1:1 ratio of nitrate and ammonium as nitrogen source (N1A1); Fe-sufficient treatment (+ Fe), Fe-deficient treatment (-Fe). Values represent the mean ± SE of three biological replicates (*n* = 3), Significant differences between treatments for each rootstock are indicated with different letters (α < 0.05, Welch ANOVA and Tukey or Games-Howell post hoc test, *N* = 3 per treatment and rootstock). **A**) *VviFRO2* (*Vitvi16g01090*); **B**) *VviAHA2* (*Vitvi11g01208*); **C**) *VviIRT1*(*Vitvi10g01358*); **D**) *VviIREG1* (*Vitvi08g02084*); **E**) *VviOPT3* (*Vitvi10g00247*); **F**) *VviVIT1* (*Vitvi01g00512*); **G**) *VviYSL6* (*Vitvi14g01520*); **H**) *VvibHLH38/39* (*Vitvi13g01037*); **I**) *VviFerritin* (*Vitvi06g01761*); **J**) *VviNR2* (*Vitvi18g00326*); **K**) *VviNRT 1:2* (*Vitvi01g00921*); **L**) *VviZAT11* (*Vitvi06g01682*)

To analyse the effect of Fe deficiency on Fe uptake and transport, a list of twelve genes involved in Fe uptake (*FRO2, IRT1, AHA2*); Fe transport (*IREG3, OPT3, VIT1, YSL6*); transcription factors involved in the regulation of *Strategy I* genes (*bHLH38/39*); Fe storage (*FERRITIN*); N uptake and transport (*NR2, NRT1:2*); genes involved in response to oxidative stress (*CAT2*); and genes involved in the regulation of root development (*ZAT11*) was compiled from the RNA-Seq analysis (Fig. 9A-L).

Corresponding to FCR activity (Fig. 4A), the expression of the *ferric reduction oxidase 2* (*FRO2*) gene was not significantly influenced by treatments in both rootstocks, but by trend higher values were observed in Fercal, while no significant change was observed with 3309 C (Fig. 9A). The rootstock genotype nor the treatment had an influence on the expression of the *iron-regulated transporter 1* (*IRT1*) but in both rootstocks enhanced values were observed with N1A0/-Fe treatment (Fig. 9C). The expression of the plasma membrane ATPase gene (*AHA2*) was enhanced under N1A0/-Fe treatment with 3309 C, while no significant change was determined with Fercal (Fig. 9B). In Fe deficiency, *bHLH38/39* significantly increased expression in both rootstocks with N1A0 treatment (Fig. 9H). The RNASeq results also showed that genes such as *iron-regulated protein 3* (*IREG3*), *oligopeptide transporter 3* (*OPT3*), and *yellow stripe-like 6* (*YSL6*) mostly showed a trend to increase their expression in both rootstocks under Fe deficiency conditions. These genes have been reported to be involved in the Fe translocation from roots to shoots under Fe-deficient conditions [70–72], while the *vacuolar iron transporter 1* (*VIT1*) was only by trend enhanced in Fercal with N1/A1-Fe (Fig. 9F). The expression of ferritin was reduced in both rootstocks in Fe-deficient roots (Fig. 9I). Related to the available N forms, *nitrate reductase 2* (*NR2*) was much higher expressed under control condition with N1A0 as compared to N1A1 in both rootstocks (Fig. 9J). When a mix of nitrate and ammonium was available, the expression in Fe-sufficient conditions (N1A1/+Fe) was much lower, than under Fe-deficient conditions (N1A1/-Fe). Such a difference in *NR2* expression was not observed between N1A0/-Fe and N1A0/+Fe, as the expression in Fe-sufficient and Fe-deficient conditions was very similar. *Nitrate transporter 1:2* (*NRT1:2*) expression was higher under N1A1/-Fe treatment than under N1A1/+Fe treatment in Fercal with a similar trend in 3309 C (Fig. 9K), whereas it was higher under N1A0/-Fe treatment than under N1A0/+Fe only in Fercal. Furthermore, *Zinc finger protein 11* (*ZAT11*), a transcriptional regulator that positively regulates primary root growth was more expressed in Fercal plants compared to 3309 C under N1A1/-Fe treatment (Fig. 9L).

## Discussion

Nitrogen and Fe are growth-limiting factors in many fruit crops due to their involvement in a variety of vital plant processes (e.g., chlorophyll synthesis, electron transfer, and photosynthesis), therefore, Fe deficiency can be a serious problem for vineyards cultivated on soils with scarce Fe bioavailability. In the present work, we sought to better understand how N forms and Fe acquisition interact to affect the physiological, biochemical, and molecular response mechanisms of two grapevine rootstocks. Thereby we aim to shed more light on the mechanisms of Fe homeostasis in grapevine rootstocks with different susceptibility to Fe chlorosis.

### Differences in symptom severity between rootstock genotypes reflect different deficiency response strategies in roots

It is well known that grapevine rootstocks differ in their tolerance to Fe chlorosis, although the mechanisms of these differences in susceptibility are not well studied [48, 73]. Our results provide some additional information to explain the different genotype response to Fe deficiency, which seems to be a combination of intrinsic factors of gene regulation and environmental influences, such as the form of available N. The pH of the nutrient solution was strongly influenced by the form of N in the nutrient solution, as the presence of ammonium reduced the pH values (more acidic), while nitrate slightly increased the pH value, this effect has already been observed in previous studies [74, 75]. The rhizosphere acidification is a well-known mechanism to enhance the availability of some nutrients, especially of Fe [76]. Nitrate-fed plants seems to suffered more from Fe deficiency exhibiting severe symptoms, low total chlorophyll contents, and low efficiency of photosystem II (FvFm). Although, the Two-Way ANOVA for the total chlorophyll content in both rootstock leaves showed no significant interaction between nitrogen forms and iron availability (Nforms * iron avail − 3309 C: F(1, 19) = 1.589, *p* = 0.226; Fercal: F(1, 18) = 1.988, *p* = 0.179), while results for FvFm revealed a significant interaction for 3309 C (F(1, 11) = 60.985, *p* = 0.001) and a minor result for Fercal (F(1, 11) = 8.303, *p* = 0.020), the observed trend for both cultivars is very similar. The mechanism behind this could be the high requirement of Fe for nitrate assimilation, as Fe contributes as a metal co-factor in enzymes of the nitrate reductive assimilatory pathway [77]. Lower symptom severity was observed when both N forms were supplied (N1A1), this could reflect that NH_4_^+^ has positive effects on Fe uptake and increases Fe root-to-shoot translocation under Fe starvation [78, 79]. Apart from the N treatment, the acidification was higher with the tolerant rootstock Fercal than with the susceptible genotype 3309 C under Fe deficiency, which could not be explained by the expression of the *AHA2* gene in root tips. Only 3309 C increased the expression level of the *AHA2* gene significantly in the presence of only nitrate. However, other factors could appear to be the causes of these changes in the pH value of the nutrient solutions, including nutrients uptake and release of root exudates [80].

Rhizosphere acidification could be one factor that differentiated the response of the rootstock genotypes under Fe deficiency, Fe uptake and root exudation could be a second one. To compensate for the low availability of Fe in soil, *Strategy I* plants increase the activity of the root ferric chelate reductase enzyme [81]. Although results were not significant, the same pattern was observed for FRC activity and expression of *FRO2* gene in Fercal, which increased both under Fe deficiency conditions, a trend not at all observed with 3309 C. Similar increases in root FCR activity have been observed in other grapevine rootstocks (Ramsey and 140 Ruggeri) in response to Fe deprivation [56, 82]. In terms of the effect of N forms on FCR activity, an interaction could only be observed for 3309 C in a Two-Way ANOVA (Nforms * iron avail − 3309 C: F(1, 11) = 16.727, *p* = 0,003; Fercal: F(1, 11) = 0.623, *p* = 0,453), although previous studies hint towards an indirect influence of N forms on FCR activity by influencing apoplastic and rhizosphere pH [81, 83].

Additionally, in various plant species exposed to Fe deficiency, increasing biosynthesis of organic acids in root tips has been shown as a strategy to mobilize Fe and increase nutrient acquisition by plants [10, 84, 85]. Indeed, also the values obtained in this study varied between rootstocks, while 3309 C considerably increased the accumulation of all organic acids in root tips with Fe deficiency, an interaction for the factors rootstock and iron availability as determined by Tow-Way ANOVA was only obtained for malic and citric acid for 3309 C (malic acid: F(1, 23) = 6.158, *p* = 0.022; citric acid: F(1, 23) = 9.766, *p* = 0.005,), while no interaction was determined for Fercal. The variability of grapevine rootstocks in organic acid’s accumulation under Fe-limiting conditions is known [73, 86], but the consequences for Fe uptake are not well studied.

In conclusion, we observed a difference in response between both rootstocks: 3309 C invests in organic acid’s accumulation in root tips, while Fercal seems to follow a strategy to enhance the ferric reductase enzyme activity in root cells. The functional consequences of these responses are beyond the scope of this study but could be an intriguing angle to pursuit in order to decipher the differences in symptom severity.

### Root morphology adaptations under Fe deficiency as a factor for nutrient uptake and translocation

An extensive root system is an important assurance for efficient nutrient uptake. In response to Fe deficiency, several plants can increase the surface area of ​​the root system [87]. In our experiment, Fercal enhanced the root biomass, associated with an increased root growth, when both N forms were supplied (N1A1/-Fe), which was not observed in 3309 C. In parallel, the expression of the C2H2-zinc finger protein *ZAT11* gene was strongly enhanced in Fercal under treatment N1A1/-Fe. In Arabidopsis, the C2H2-zinc finger protein family has been demonstrated to be essential for several important cellular functions, including transcriptional regulation, development, and stress responses [88]. Among these genes, *ZAT11* has been reported to have an impact on root development [89, 90]. Several plant hormones have been reported to be involved in regulating responses to Fe deficiency. It has been suggested that auxin is involved in the induction of modifications in root architecture, such as increasing root branching and the density and length of lateral roots, which could be adaptive strategies used by several plant species to facilitate Fe acquisition [16, 91], indicating that plants under low Fe availability reduced the aboveground biomass more than that of roots [92]. A shift in the root-shoot ration was not observed in our results. Nevertheless, the enhanced root biomass including an increase in root branching of Fercal could contribute to the enhancement of FCR activity in roots, by simply increasing the number of active root tips. Future studies need to quantify the number of root tips to conclude the size effect of morphological adaptations in combination with biochemical plant responses.

#### Plant Fe content is modulated by Fe availability, N form, and Fe translocation

The severity of Fe chlorosis symptoms under Fe deficiency was affected by the rootstock genotype and the N form availability. Iron contents in leaves and roots are affected not just by the Fe availability but also by the supplied N forms partially independent of the rootstock genotype. With regard to N form, 3309 C plants treated with nitrate as the sole N form (which had severe chlorosis symptoms) showed a higher accumulation of Fe in roots under Fe sufficient conditions (mean values: N1A0 + Fe = 3367.8 ± 679.0, N1A1 + Fe = 378.8 ± 42.9; Welch´s F(1, 4.032) = 96.365, *p* = 0.001), while values in Fercal were only slightly affected by the N form (mean values: N1A0 + Fe = 4058.3 ± 770.0, N1A1 + Fe = 2369.6 ± 685.0; Welch´s F(1, 5.750) = 11.585, *p* = 0.015). Plants fed with mixed N nutrition were characterized by higher Fe content in older leaves (3309 C: mean values: N1A0 + Fe = 82.1 ± 2.50, N1A1 + Fe = 155.6 ± 10.8; Welch´s F(1, 2,206) = 131.754, *p* = 0.005; Fercal: mean values: N1A0 + Fe = 61.9 ± 12.3, N1A1 + Fe = 144.5 ± 13.5; Welch´s F(1, 3.970) = 61.339, *p* = 0.001). These findings could indicate that the combined ammonium and nitrate supply increased the Fe translocation from roots to mainly old leaves, while nitrate supply enhanced Fe retention in the root tissues. Similar results, higher Fe accumulation in roots with nitrate nutrition, were obtained with other crop species in previous studies [27, 93]. These authors proposed a high pH value in the root apoplast resulting in a limitation of the reduction of Fe ^III^-chelates by the FCR enzyme as a possible mechanism.

In return, Fe deficiency probably affects nitrate assimilation, our results showed that nitrate reductase *NR2* gene tended to decrease it expression under N1A0 treatment in both rootstocks. Similar effect of Fe deficiency on the reduction of *NR2* gene expression was already observed in Fe-deficient plants grown in nitrate as a sole source of N such as cucumber [94], tomato [95], and grapevine [2]. This reduction is potentially due to the decreased expression of genes encoding Fe-containing enzymes (e.g., NR and NiR) [94].

Grapevine rootstocks in our experiment affected the relation between Fe content in young leaves and their total chlorophyll content. Although Fe is essential for chlorophyll biosynthesis [96], the observed phenotype and the changes in chlorophyll could reflect the Fe requirement for nitrate assimilation, as recently proposed [77]. The chlorophyll content of plants under treatment N1A1/-Fe were less affected compared to plants supplied with nitrate as the sole N source (3309 C: mean values: N1A0/-Fe = 2.75 ± 0.38, N1A1/-Fe = 5.86 ± 0.78; Welch´s F(1, 5.808) = 12.907, *p* = 0.012; Fercal: mean values: N1A0/-Fe = 3.23 ± 0.35, N1A1/-Fe = 6.01 ± 0.78; Welch´s F(1, 5.560) = 10.480, *p* = 0.020). Although the N content in leaves was only marginal affected by the treatments (Table S1), we observed lowest contents in both rootstocks with treatment N1A0/-Fe (OL 3309 C: 2.58 ± 0.03; OL Fercal: 2.28 ± 0.11), while values were slightly higher when both N forms were available OL 3309 C: 3.17 ± 0.18; OL Fercal: 2.95 ± 0.22). Plant grown under Fe-sufficient conditions showed a trend of slightly higher N contents in old leaves, although no significant differences were determined, maybe supporting the crucial need of Fe when plants assimilate nitrate [92, 94].

Many studies confirmed a considerable decrease in the photosynthesis in various fruit tree species under Fe deficiency stress [11, 97, 98]. Despite the loss of chlorophyll, chlorotic leaves in our study did not exhibit a noticeable decrease in transpiration rate or stomatal conductance within the experimental timeframe. These outcomes are consistent with those that have been reported for grapevine [73] and sugar beet [99]. Contrarily the efficiency of PSII was affected by Fe deficiency, the ratio of variable to maximum fluorescence of PSII (Fv/Fm) was significantly limited in both rootstocks, although the effect on 3309 C plants was more severe. The Tow-Way ANOVA confirmed under Fe-deficient conditions a significant influence of the rootstock (F(1, 11) = 56.890, *p* < 0.001) and the N forms (F(1, 11) = 8.506, *p* = 0.019), while the interaction effect was small (F(1, 11) = 5.627, *p* = 0.045). This decrease may be due to the reduced energy transfer from the PSII to the reaction centers [100], probably due to reduced chlorophyll concentrations caused by the absence of Fe [51, 73, 101]. A growth limitation could be the consequence, as an appropriate NO_3_^−^/ NH_4_^+^ ratio has been largely reported in the literature to have a positive effect on sustaining plant growth and increasing both biomass accumulation and quality in different crops including grapevine [102–104].

### Complex phytohormonal and transcriptional response regulation due to Fe deficiency and N forms

**RNASeq** analyses of root tips enabled us to identify similarities and dissimilarities in response reactions between rootstocks and treatments. GO pathways associated with DEGs indicated differences in Fercal and 3309 C responses when grown under different N forms supply. Under N1A0 treatment, the genes involved in iron ion binding pathway were overexpressed in both rootstocks. These results indicated that both rootstocks tended to store Fe in their roots when supplied solely with nitrate over extended periods of Fe starvation, resulting in obvious chlorosis symptoms on young leaves [105]. Moreover, both rootstocks exhibited several up-regulated genes involved in various stress responses pathways. Similar results were obtained by [106] in the case of citrus rootstocks grown under natural Fe-deficiency conditions, which appear to be associated with general responses to Fe deficiency, most likely by preventing the negative effects of reactive oxygen species damages [107]. In contrast, several genes involved in phytohormones pathways were significantly down-regulated in the auxin metabolic pathway in Fercal and in the ethylene signalling pathway in 3309 C. These two phytohormones have been widely demonstrated to be involved in regulating the responses to Fe deficiency by stimulating plant root growth and increasing the expression of genes involved in Fe acquisition in *Strategy I* plants [108, 109].

On the other hand, the equal supply of both forms of N (N1A1) showed a positive effect on the expression of genes that help in alleviating Fe deficiency stress. Notably, DEGs involved in xyloglucan:xyloglucosyl transferase activity were overexpressed in both rootstocks. This subcategory includes genes corresponding to cell wall modifying enzymes which are essential for the expansion and reconstruction of the cell wall [110]. Furthermore, several genes with significantly increased expression in the trehalose biosynthetic and sucrose synthase pathways were detected in 3309 C plants. Multiple studies have shown an involvement of sucrose in regulating the level of Fe deficiency by enhancing auxin levels in roots and increasing the expression of Fe-uptake related genes (*FRO2* and *IRT1*) [111]. In addition, trehalose metabolism has been reported to play a crucial role in protecting cellular structures from abiotic stresses [112] and to function as a signal of sucrose levels [113].

Several phytohormones, such as auxin [45], abscisic acid (ABA) [46], ethylene [114], and cytokinin [47] are considered to play either a positive or a negative role in regulating plant responses to Fe deficiency. According to reports, auxin can cause the plant root system to branch by promoting the growth of lateral roots [115]. The overexpression of auxin primary response genes within Aux/IAA and SAUR families in plants grown under Fe deficiency with both N forms present supports those observations of the increase in root biomass accumulation, including an apparent (not-quantified) enhanced branching, which was more evident with Fercal rootstock.

Although Fe has been considered to have limited mobility towards young leaves, recent research has demonstrated that in conditions of Fe deprivation, Fe can be partially remobilized from the root cell wall to the sink organs [78, 116]. Abscisic acid has been also considered to have a positive impact on Fe deficiency symptoms by promoting Fe transportation and translocation [117]. In this work, an Fe deficiency-induced expression of genes involved in the ABA signalling pathway (PCAR, PYL, PP2CA, SnRK2) was observed with both rootstocks when supplied with both N forms. Low nitrate levels have been shown to increase plant ABA content, which promotes the Fe remobilization from cell walls and its translocation from root to shoot [118]. As a consequence, a balanced supply of N with different forms could support the stress response of grapevine rootstocks under Fe deficiency.

A high number of genes were differentially expressed under the applied treatments, involving several molecular functions and biological processes as determined by the GO enrichment analysis. In the first step, we focused on genes involved in Fe uptake, Fe translocation, and their regulation. Previous research showed a strong connection between Fe uptake in roots and Fe deficiency chlorosis, suggesting the involvement of Fe uptake genes (*AHA2, IRT1, FRO2*) in the regulation of Fe acquisition [119]. Expression of all of these genes was also induced by trend in our study, in particular, *FRO2* with rootstock Fercal. As a consequence of the higher pH values under N1A0 treatment, 3309 C aimed to contain these pH increases by enhancing the expression of *AHA2* under Fe deficiency. Similarly, under the same treatment, the expression of *IRT1* was increased by trend in both rootstocks, suggesting a higher sensitivity to scarce Fe availability when N is applied only in nitrate form [39]. On the other hand, the tolerant rootstock Fercal exhibited by trend a higher ability to increase the expression of *FRO2*, especially in N1A1/-Fe treatment, which can also be explained by the ability of this rootstock to increase the root system under this treatment providing more sites for FCR activity [92]. However, with the current knowledge, it is not clear how much this response is participating in the tolerance of Fercal to Fe deficiency.

Plant Fe homeostasis is strongly regulated by transcription factors of the bHLH family [120]. Our research also revealed that *bHLH38/3*9 was more expressed under Fe deficiency with nitrate as the sole source of N compared with nitrate: ammonium supply (1:1). This finding supports previous reports that suggested a direct participation of this gene in the regulation of Fe deficiency response [39].

Ferritins have been characterized to be involved in the Fe storage in several plants, which can be released when necessary [30]. In line with previous studies, both rootstocks had varied degrees of down-regulation of the ferritin genes under Fe-deficient conditions, along with a trend to increase the expression of genes linked to Fe transport and mobilization, such as *IREG3, OPT3, VIT1*, and *YSL6* [11, 30, 121]. This outcome highlights the value of ferritin storage capacity in maintaining Fe concentration in all plant parts. A quantification of ferritin in different tissues would help to understand its role in Fe redistribution within plants.

## Conclusion

In summary, grapevine rootstocks differ in their tolerance to Fe chlorosis and have different abilities to modify their growth and physiology to adapt to Fe absence. Fercal demonstrates a higher ability to promote root growth and activities involved in *Strategy I* plant responses under Fe-deficient conditions. On the other hand, the supplied N forms appear to have a great influence on Fe uptake and remobilization. Under Fe-deficient conditions, the interactions between nitrate and Fe affect plant productivity and result in more severe chlorosis symptoms, most probably due to the effect of nitrate acquisition in lowering Fe solubility. In return, the low availability of Fe results in lower nitrate assimilation. However, it is still unclear how nitrate interferes with Fe acquisition at the molecular level. In contrast, the addition of ammonium to the N supplied appears to be able to alleviate Fe deficiency through either or a combination of the following hypothesis: (i) improving Fe availability by its impact on lowering medium pH; (ii) increasing the root apparatus ramification, providing more sites for FCR enzyme; (iii) reducing the demand of Fe required for nitrate reduction process; (iv) improving the availability of soluble Fe in roots by increasing the release of Fe from the cell wall; (v) and enhancing Fe reutilization and transport from source to sink organs. Furthermore, the expression of genes involved in the auxin and abscisic acid signal transduction pathways is significantly induced in a response to limited Fe availability when both forms of N are equally supplied, suggesting a possible role of the addition of ammonium in the activation of these phytohormones, which is reported to have a positive effect on alleviating Fe deficiency stress.

### Electronic supplementary material

Below is the link to the electronic supplementary material.

Supplementary Material 1

Supplementary Material 2

## Data Availability

The datasets of physiological and analytical methods generated and/or analysed during the current study are available in the Zenodo repository of the University of Natural Resources and Life Sciences, Vienna, with DOI: 10.5281/zenodo.10470469. The RNA-seq raw dataset analyzed in this study is deposited in NCBI SRA with BioProject accession number PRJNA1068036 (https://www.ncbi.nlm.nih.gov/bioproject/PRJNA1068036).

## References

[1] Ksouri K, Mahmoudi R, Gharsalli M, Lachaâl M (2015). Physiological responses of native Tunisian grapevines and some rootstocks to direct iron deficiency. Vitis.

[2] Zebec V, Lisjak M, Jović J, Kujundžić T, Rastija D, Lončarić Z (2021). Vineyard fertilization management for iron deficiency and chlorosis prevention on carbonate soil. Hortic.

[3] Guo G, Xiao J, Jeong BR (2022). Iron source and medium pH affect nutrient uptake and pigment content in Petunia hybrida ‘Madness red’cultured in vitro. Int J Mol Sci.

[4] Hsieh E-J, Waters BM (2016). Alkaline stress and iron deficiency regulate iron uptake and riboflavin synthesis gene expression differently in root and leaf tissue: implications for iron deficiency chlorosis. J Exp Bot.

[5] Briat J-F, Dubos C, Gaymard F (2015). Iron nutrition, biomass production, and plant product quality. Trends Plant Sci.

[6] Tagliavini M, Rombola AD (2001). Iron deficiency and chlorosis in orchard and vineyard ecosystems. Eur J Agron.

[7] Rustioni L, Grossi D, Brancadoro L, Failla O (2017). Characterization of iron deficiency symptoms in grapevine (Vitis spp.) leaves by reflectance spectroscopy. Plant Physiol Biochem.

[8] Rossdeutsch L, Schreiner RP, Skinkis PA, Deluc L (2021). Nitrate uptake and transport properties of two grapevine rootstocks with varying vigor. Front Plant Sci.

[9] Sabir A, Ekbic H, Erdem H, Tangolar S (2010). Response of four grapevine (Vitis spp.) genotypes to direct or bicarbonate-induced iron deficiency. Span J Agric.

[10] Covarrubias JI, Rombolà AD (2015). Organic acids metabolism in roots of grapevine rootstocks under severe iron deficiency. Plant Soil.

[11] Vannozzi A, Donnini S, Vigani G, Corso M, Valle G, Vitulo N, Bonghi C, Zocchi G, Lucchin M (2017). Transcriptional characterization of a widely-used grapevine rootstock genotype under different iron-limited conditions. Front Plant Sci.

[12] Nikolic M, Kastori R (2000). Effect of bicarbonate and Fe supply on Fe nutrition of grapevine. J Plant Nutr.

[13] Ramírez L, Graziano M, Lamattina L (2008). Decoding plant responses to iron deficiency: is nitric oxide a central player?. Plant Signal Behav.

[14] Młodzińska-Michta E. Abiotic factors determine the root system architecture – review and update. Acta Soc Bot Pol. 2023; 92(1).

[15] Boamponsem GA, Leung DW, Lister C (2017). Insights into resistance to Fe deficiency stress from a comparative study of in vitro-selected novel Fe-efficient and Fe-inefficient potato plants. Front Plant Sci.

[16] Jin CW, Chen WW, Meng ZB, Zheng SJ (2008). Iron deficiency-induced increase of root branching contributes to the enhanced root ferric chelate reductase activity. J Integr Plant Biol.

[17] Siminis CI, Stavrakakis MN (2008). Iron induces root and leaf ferric chelate reduction activity in grapevine rootstock 140 Ruggeri. Hortic Sci.

[18] Gutiérrez-Gamboa G, Alañón-Sánchez N, Mateluna-Cuadra R, Verdugo-Vásquez N (2020). An overview about the impacts of agricultural practices on grape nitrogen composition: current research approaches. Food Res Int.

[19] Hachiya T, Sakakibara H (2017). Interactions between nitrate and ammonium in their uptake, allocation, assimilation, and signaling in plants. J Exp Bot.

[20] Mengel K (1994). Iron availability in plant tissues-iron chlorosis on calcareous soils. Plant Soil.

[21] Buoso S, Tomasi N, Said-Pullicino D, Arkoun M, Yvin J-C, Pinton R, Zanin L (2021). Characterization of physiological and molecular responses of Zea mays seedlings to different urea-ammonium ratios. Plant Physiol Biochem.

[22] Yin H, Li B, Wang X, Xi Z (2020). Effect of ammonium and nitrate supplies on nitrogen and sucrose metabolism of Cabernet Sauvignon (Vitis vinifera Cv). J Sci Food Agric.

[23] Datta S. A brief Note on Iron Deficiency in Plants and its Correction. 2019.

[24] Mengel K, Geurtzen G (1988). Relationship between iron chlorosis and alkalinity in Zea mays. Physiol Plant.

[25] Kosegarten H, Schwed U, Wilson G, Mengel K (1998). Comparative investigation on the susceptibility of faba bean (Vicia faba L.) and sunflower (Helianthus annuus L.) to iron chlorosis. J Plant Nutr.

[26] Covarrubias JI, Pisi A, Rombolà A (2014). Evaluation of sustainable management techniques for preventing iron chlorosis in the grapevine. Aust J Grape Wine Res.

[27] Zou C, Shen J, Zhang F, Guo S, Rengel Z, Tang C (2001). Impact of nitrogen form on iron uptake and distribution in maize seedlings in solution culture. Plant Soil.

[28] De la Peña M, Marín-Peña AJ, Urmeneta L, Coleto I, Castillo-González J, van Liempd SM, Falcón-Pérez JM, Álvarez-Fernández A, González-Moro MB, Marino D (2022). Ammonium nutrition interacts with iron homeostasis in Brachypodium distachyon. J Exp Bot.

[29] Zhu XF, Dong XY, Wu Q, Shen RF (2019). Ammonium regulates Fe deficiency responses by enhancing nitric oxide signaling in Arabidopsis thaliana. Planta.

[30] Zhang X, Zhang D, Sun W, Wang T. The adaptive mechanism of plants to Iron Deficiency via Iron Uptake, Transport, and Homeostasis. Int J Mol Sci. 2019; 20(10). 10.3390/ijms20102424 PMC6566170 31100819

[31] Stefanello LO, Schwalbert R, Schwalbert RA, De Conti L, de Souza Kulmann MS, Garlet LP, Silveira MLR, Sautter CK, de Melo GWB, Rozane DE (2020). Nitrogen supply method affects growth, yield and must composition of young grape vines (Vitis vinifera L. Cv Alicante Bouschet) in southern Brazil. Sci Hortic.

[32] Borrero C, Trillas M, Delgado A, Avilés M (2012). Effect of ammonium/nitrate ratio in nutrient solution on control of Fusarium wilt of tomato by Trichoderma Asperellum T34. Plant Pathol.

[33] Curie C, Mari S (2017). New routes for plant iron mining. New Phytol.

[34] Kobayashi T, Nishizawa NK (2012). Iron uptake, translocation, and regulation in higher plants. Annu Rev Plant Biol.

[35] Jeong J, Merkovich A, Clyne M, Connolly EL (2017). Directing iron transport in dicots: regulation of iron acquisition and translocation. Curr Opin Plant Biol.

[36] Tsai HH, Schmidt W (2017). Mobilization of iron by plant-borne coumarins. Trends Plant Sci.

[37] Sahin O, Gunes A, Taskin MB, Inal A (2017). Investigation of responses of some apple (Mallus x Domestica Borkh.) Cultivars grafted on MM106 and M9 rootstocks to lime-induced chlorosis and oxidative stress. Sci Hortic.

[38] Fu L, Zhu Q, Sun Y, Du W, Pan Z, Sa P (2017). Physiological and transcriptional changes of three citrus rootstock seedlings under iron deficiency. Front Plant Sci.

[39] Gao F, Robe K, Gaymard F, Izquierdo E, Dubos C (2019). The transcriptional control of iron homeostasis in plants: a tale of bHLH transcription factors?. Front Plant Sci.

[40] Santi S, Schmidt W (2009). Dissecting iron deficiency-induced proton extrusion in Arabidopsis roots. New Phytol.

[41] Robinson NJ, Procter CM, Connolly EL, Guerinot ML (1999). A ferric-chelate reductase for iron uptake from soils. Nature.

[42] Eide D, Broderius M, Fett J, Guerinot ML (1996). A novel iron-regulated metal transporter from plants identified by functional expression in yeast. Proc Natl Acad Sci.

[43] Colangelo EP, Guerinot ML (2004). The essential basic helix-loop-helix protein FIT1 is required for the iron deficiency response. Plant Cell.

[44] Lucena C, Porras R, García MJ, Alcántara E, Pérez-Vicente R, Zamarreño ÁM, Bacaicoa E, García-Mina JM, Smith AP, Romera FJ. Ethylene and Phloem signals are involved in the regulation of responses to Fe and P deficiencies in roots of strategy I plants. Front Plant Sci. 2019; 10. 10.3389/fpls.2019.01237 PMC6795750 31649701

[45] Sun H, Feng F, Liu J, Zhao Q. The Interaction between Auxin and nitric oxide regulates Root Growth in response to Iron Deficiency in Rice. Front Plant Sci. 2017; 8. 10.3389/fpls.2017.02169 PMC5743679 29312409

[46] Lei GJ, Zhu XF, Wang ZW, Dong F, Dong NY, Zheng SJ (2014). Abscisic acid alleviates iron deficiency by promoting root iron reutilization and transport from root to shoot in Arabidopsis. Plant Cell Environ.

[47] Séguéla M, Briat JF, Vert G, Curie C (2008). Cytokinins negatively regulate the root iron uptake machinery in Arabidopsis through a growth-dependent pathway. Plant J.

[48] Jiménez S, Gogorcena Y, Hévin C, Rombola AD, Ollat N (2007). Nitrogen nutrition influences some biochemical responses to iron deficiency in tolerant and sensitive genotypes of Vitis. Plant Soil.

[49] Molina J, Covarrubias JI (2019). Influence of nitrogen on physiological responses to bicarbonate in a grapevine rootstock. J Soil Sci Plant Nutr.

[50] Hoagland D, Arnon D. The water-culture method for growing plants without soil. Circular. California agricultural experiment station, 1950; 347 (2nd edit).

[51] Cambrollé J, García JL, Figueroa ME, Cantos M (2014). Physiological responses to soil lime in wild grapevine (Vitis vinifera ssp. sylvestris). Environ Exp Bot.

[52] Pouget R, Ottenwaelter M (1978). Etude De L’adaptation De Nouvelles variétés De Porte-Greffes à des sols très chlorosants. Conn Vigne Vin.

[53] Rouse JW, Haas RH, Schell JA, Deering DW (1974). Monitoring vegetation systems in the Great Plains with ERTS. NASA Spec Publ.

[54] Daughtry CS, Walthall C, Kim M, De Colstoun EB, McMurtrey Iii J (2000). Estimating corn leaf chlorophyll concentration from leaf and canopy reflectance. Remote Sens Environ.

[55] Hiscox J, Israelstam G (1979). A method for the extraction of chlorophyll from leaf tissue without maceration. Can J Bot.

[56] Marastoni L, Lucini L, Miras-Moreno B, Trevisan M, Sega D, Zamboni A, Varanini Z (2020). Changes in physiological activities and root exudation profile of two grapevine rootstocks reveal common and specific strategies for Fe acquisition. Sci Rep.

[57] Savoi S, Herrera JC, Forneck A, Griesser M (2019). Transcriptomics of the grape berry shrivel ripening disorder. Plant Mol Biol.

[58] Simons A. FastQC. A quality control tool for high throughput sequence data (Online). 2010. Available online at: http://www.bioinformatics.babraham.ac.uk/projects/fastqc/.

[59] Okonechnikov K, Conesa A, Qualimap FG-A. Qualimap2: Advanced multi-sample quality control for high-throughput sequencing data. Bioinform 2016; 32. 10.1093/bioinformatics/btv566 PMC4708105 26428292

[60] Ewels P, Magnusson M, Lundin S, Käller M (2016). MultiQC: summarize analysis results for multiple tools and samples in a single report. Bioinform.

[61] Dobin A, Davis CA, Schlesinger F, Drenkow J, Zaleski C, Jha S, Batut P, Chaisson M, Gingeras TR (2013). STAR. Ultrafast universal RNA-seq aligner. Bioinform.

[62] Liao Y, Smyth GK, Shi W (2014). featureCounts: an efficient general purpose program for assigning sequence reads to genomic features. Bioinform.

[63] Götz S, García-Gómez JM, Terol J, Williams TD, Nagaraj SH, Nueda MJ, Robles M, Talón M, Dopazo J, Conesa A (2008). High-throughput functional annotation and data mining with the Blast2GO suite. Nucleic Acids Res.

[64] Ge SX, Son EW, Yao R (2018). iDEP: an integrated web application for differential expression and pathway analysis of RNA-Seq data. BMC Bioinform.

[65] Bonnot T, Gillard MB, Nagel DH. A simple protocol for informative visualization of enriched gene ontology terms. Bio-protoc. 2019;e3429–3429.

[66] Love M, Anders S, Huber W (2014). Differential analysis of count data–the DESeq2 package. Genome Biol.

[67] Ye YQ, Luo HY, Li M, Zhang JJ, Cao GQ, Lin KM, Lin SZ, Xu SS (2019). Potassium ameliorates iron deficiency by facilitating the remobilization of iron from root cell walls and promoting its translocation from roots to shoots. Plant Soil.

[68] Kanwar P, Baby D, Bauer P (2021). Interconnection of iron and osmotic stress signalling in plants: is FIT a regulatory hub to cross-connect abscisic acid responses?. Plant Biol.

[69] Chen WW, Yang JL, Qin C, Jin CW, Mo JH, Ye T, Zheng SJ (2010). Nitric oxide acts downstream of auxin to trigger root ferric-chelate reductase activity in response to iron deficiency in Arabidopsis. Plant Physiol.

[70] Kim LJ, Tsuyuki KM, Hu F, Park EY, Zhang J, Iraheta JG, Chia JC, Huang R, Tucker AE, Clyne M (2021). Ferroportin 3 is a dual-targeted mitochondrial/chloroplast iron exporter necessary for iron homeostasis in Arabidopsis. Plant J.

[71] Wang C, Wang X, Li J, Guan J, Tan Z, Zhang Z, Shi G (2022). Genome-wide identification and transcript analysis reveal potential roles of oligopeptide transporter genes in iron deficiency induced cadmium accumulation in peanut. Front Plant Sci.

[72] Kobayashi T, Nozoye T, Nishizawa NK (2019). Iron transport and its regulation in plants. Free Radic Biol Med.

[73] Covarrubias JI, Retamales C, Donnini S, Rombolà AD, Pastenes C (2016). Contrasting physiological responses to iron deficiency in Cabernet Sauvignon grapevines grafted on two rootstocks. Sci Hortic.

[74] Wang J, Tu X, Zhang H, Cui J, Ni K, Chen J, Cheng Y, Zhang J, Chang SX (2020). Effects of ammonium-based nitrogen addition on soil nitrification and nitrogen gas emissions depend on fertilizer-induced changes in pH in a tea plantation soil. Sci Total Environ.

[75] Feng H, Fan X, Miller AJ, Xu G (2020). Plant nitrogen uptake and assimilation: regulation of cellular pH homeostasis. J Exp Bot.

[76] Molnár Z, Solomon W, Mutum L, Janda T (2023). Understanding the mechanisms of Fe deficiency in the rhizosphere to promote plant resilience. Plants.

[77] Bian Z, Wang Y, Zhang X, Li T, Grundy S, Yang Q, Cheng R. A review of Environment effects on Nitrate Accumulation in Leafy vegetables grown in controlled environments. Foods 2020; 9(6). 10.3390/foods9060732 PMC7353485 32503134

[78] Zhu CQ, Zhang JH, Zhu LF, Abliz B, Zhong C, Bai ZG, Hu WJ, Sajid H, James AB, Cao XC (2018). NH4 + facilitates iron reutilization in the cell walls of rice (Oryza sativa) roots under iron-deficiency conditions. Environ Exp Bot.

[79] Marino D, Moran JF. Can ammonium stress be positive for plant performance? Front Plant Sci. 2019; 10. 10.3389/fpls.2019.01103 PMC6771378 31608080

[80] Chen Y-T, Wang Y, Yeh K-C (2017). Role of root exudates in metal acquisition and tolerance. Curr Opin Plant Biol.

[81] Poonnachit U, Darnell R (2004). Effect of ammonium and nitrate on ferric chelate reductase and nitrate reductase in Vaccinium species. Ann Bot.

[82] Ozdemir G, Devrim B. Effects of different iron applications on root ferric chelate reductase activity of öküzgözü and boğazkere grape varieties; 2018. Conference (IENSC 2018).

[83] Zhao T, Ling HQ (2007). Effects of pH and nitrogen forms on expression profiles of genes involved in iron homeostasis in tomato. Plant Cell Environ.

[84] Mora-Córdova CP, Tolrà R, Padilla R, Poschenrieder C, Simard M-H, Asín L, Vilardell P, Bonany J, Claveria E, Dolcet-Sanjuan R (2022). Rhizosphere Acidification as the Main Trait characterizing the Differential in Vitro Tolerance to Iron Chlorosis in Interspecific Pyrus hybrids. Hortic.

[85] Kabir AH, Rahman MA, Rahman MM, Brailey-Jones P, Lee KW, Bennetzen JL (2022). Mechanistic assessment of tolerance to iron deficiency mediated by Trichoderma Harzianum in soybean roots. J Appl Microbiol.

[86] Ollat N, Laborde B, Neveux M, Diakou-Verdin P, Renaud C, Moing A (2003). Organic Acid Metabolism in roots of various grapevine (Vitis) rootstocks submitted to Iron Deficiency and Bicarbonate Nutrition. J Plant Nutr.

[87] Li G, Kronzucker HJ, Shi W. The response of the Root apex in Plant Adaptation to Iron Heterogeneity in Soil. Front Plant Sci. 2016; 7. 10.3389/fpls.2016.00344 PMC4800179 27047521

[88] Ciftci-Yilmaz S, Mittler R (2008). The zinc finger network of plants. Cell Mol Life Sci.

[89] Liu XM, An J, Han HJ, Kim SH, Lim CO, Yun DJ, Chung WS (2014). ZAT11, a zinc finger transcription factor, is a negative regulator of nickel ion tolerance in Arabidopsis. Plant Cell Rep.

[90] Xie M, Sun J, Gong D, Kong Y. The roles of Arabidopsis C1-2i subclass of C2H2-type zinc-finger transcription factors. Genes (Basel) 2019; 10(9). 10.3390/genes10090653 PMC6770587 31466344

[91] Challam C, Dutt S, Sudhakar D, Raveendran M, Buckseth T, Singh RK (2021). Increase in root branching enhanced ferric-chelate reductase activity under iron stress in potato (Solanum tuberosum). Indian J Agric Sci.

[92] Liu J, Wang J, Wang Z, Li M, Liang C, Yang Y, Li D, Wang R (2022). Alleviation of iron deficiency in pear by ammonium nitrate and nitric oxide. BMC Plant Biol.

[93] Assimakopoulou A (2006). Effect of iron supply and nitrogen form on growth, nutritional status and ferric reducing activity of spinach in nutrient solution culture. Sci Hortic.

[94] Borlotti A, Vigani G, Zocchi G (2012). Iron deficiency affects nitrogen metabolism in cucumber (Cucumis sativus L.) plants. BMC Plant Biol.

[95] Wang YH, Garvin DF, Kochian LV (2001). Nitrate-induced genes in tomato roots. Array analysis reveals novel genes that may play a role in nitrogen nutrition. Plant Physiol.

[96] Sun Y, Luo J, Feng P, Yang F, Liu Y, Liang J, Wang H, Zou Y, Ma F, Zhao T (2022). MbHY5-MbYSL7 mediates chlorophyll synthesis and iron transport under iron deficiency in Malus baccata. Front Plant Sci.

[97] Wang Y-x, Hu Y, Zhu Y-f, Baloch AW, Jia X-m, Guo A-x (2018). Transcriptional and physiological analyses of short-term Iron deficiency response in apple seedlings provide insight into the regulation involved in photosynthesis. BMC Genom.

[98] Eichert T, Peguero-Pina JJ, Gil-Pelegrín E, Heredia A, Fernández V (2010). Effects of iron chlorosis and iron resupply on leaf xylem architecture, water relations, gas exchange and stomatal performance of field-grown peach (Prunus persica). Physiol Plant.

[99] Rombolà AD, Gogorcena Y, Larbi A, Morales F, Baldi E, Marangoni B, Tagliavini M, Abadía J (2005). Iron deficiency-induced changes in carbon fixation and leaf elemental composition of sugar beet (Beta vulgaris) plants. Plant Soil.

[100] Bertamini M, Nedunchezhian N (2005). Grapevine growth and physiological responses to Iron Deficiency. J Plant Nutr.

[101] Bertamini M, Muthuchelian K, Nedunchezhian N (2002). Iron deficiency induced changes on the donor side of PS II in field grown grapevine (Vitis vinifera L. Cv. Pinot noir) leaves. Plant Sci.

[102] Zhu Y, Qi B, Hao Y, Liu H, Sun G, Chen R, Song S. Appropriate NH4+/NO3– ratio triggers Plant Growth and Nutrient Uptake of Flowering Chinese Cabbage by optimizing the pH value of nutrient solution. Front Plant Sci. 2021; 12. 10.3389/fpls.2021.656144 PMC8121088 33995453

[103] Martínez-Moreno R, Quirós M, Morales P, Gonzalez R (2014). New insights into the advantages of ammonium as a winemaking nutrient. Int J Food Microbiol.

[104] Wang P, Wang Z, Pan Q, Sun X, Chen H, Chen F, Yuan L, Mi G (2019). Increased biomass accumulation in maize grown in mixed nitrogen supply is mediated by auxin synthesis. J Exp Bot.

[105] Kosegarten H, Wilson G, Esch A (1998). The effect of nitrate nutrition on iron chlorosis and leaf growth in sunflower (Helianthus annuus L). Eur J Agron.

[106] Licciardello C, Torrisi B, Allegra M, Sciacca F, Roccuzzo G, Intrigliolo F, Recupero GR, Tononi P, Delledonne M, Muccilli V (2013). A transcriptomic analysis of sensitive and tolerant citrus rootstocks under natural iron deficiency conditions. J Am Soc Hortic Sci.

[107] Rellán-Álvarez R, Andaluz S, Rodríguez-Celma J, Wohlgemuth G, Zocchi G, Álvarez-Fernández A, Fiehn O, López-Millán AF, Abadía J (2010). Changes in the proteomic and metabolic profiles of Beta vulgaris root tips in response to iron deficiency and resupply. BMC Plant Biol.

[108] Romera FJ, García MJ, Alcántara E, Pérez-Vicente R (2011). Latest findings about the interplay of auxin, ethylene and nitric oxide in the regulation of Fe deficiency responses by Strategy I plants. Plant Signal Behav.

[109] Celletti S, Pii Y, Valentinuzzi F, Tiziani R, Fontanella MC, Beone GM, Mimmo T, Cesco S, Astolfi S (2020). Physiological responses to Fe deficiency in split-root tomato plants: possible roles of auxin and ethylene?. Agronomy.

[110] Stratilová B, Kozmon S, Stratilová E, Hrmova M (2020). Plant xyloglucan xyloglucosyl transferases and the cell wall structure: subtle but significant. Molecules.

[111] Lin XY, Ye YQ, Fan SK, Jin CW, Zheng SJ (2016). Increased sucrose accumulation regulates iron-deficiency responses by promoting auxin signaling in Arabidopsis plants. Plant Physiol.

[112] Du L, Li S, Ding L, Cheng X, Kang Z, Mao H (2022). Genome-wide analysis of trehalose-6-phosphate phosphatases (TPP) gene family in wheat indicates their roles in plant development and stress response. BMC Plant Biol.

[113] Paul MJ (2008). Trehalose 6-phosphate: a signal of sucrose status. Biochem J.

[114] Lucena C, Romera FJ, García MJ, Alcántara E, Pérez-Vicente R. Ethylene participates in the regulation of Fe Deficiency responses in Strategy I plants and in Rice. Front Plant Sci. 2015; 6. 10.3389/fpls.2015.01056 PMC4661236 26640474

[115] Jain M, Kaur N, Tyagi AK, Khurana JP (2006). The auxin-responsive GH3 gene family in rice (Oryza sativa). Funct Integr Genomics.

[116] Jin CW, You GY, He YF, Tang C, Wu P, Zheng SJ (2007). Iron Deficiency-Induced Secretion of Phenolics facilitates the reutilization of Root Apoplastic Iron in Red Clover. Plant Physiol.

[117] Zhang J-C, Wang X-F, Wang X-N, Wang F-P, Ji X-L, An J-P, Yang K, Zhao Q, You C-X, Hao Y-J (2020). Abscisic acid alleviates iron deficiency by regulating iron distribution in roots and shoots of apple. Sci Hortic.

[118] Sun WJ, Zhang JC, Ji XL, Feng ZQ, Wang X, Huang WJ, You CX, Wang XF, Hao YJ (2021). Low nitrate alleviates iron deficiency by regulating iron homeostasis in apple. Plant Cell Environ.

[119] Ye JY, Zhou M, Zhu QY, Zhu YX, Du WX, Liu XX, Jin CW (2022). Inhibition of shoot-expressed NRT1.1 improves reutilization of apoplastic iron under iron-deficient conditions. Plant J.

[120] Li X, Zhang H, Ai Q, Liang G, Yu D (2016). Two bHLH transcription factors, bHLH34 and bHLH104, regulate Iron Homeostasis in Arabidopsis thaliana. Plant Physiol.

[121] Yuan J, Li D, Shen C, Wu C, Khan N, Pan F, Yang H, Li X, Guo W, Chen B et al. Transcriptome analysis revealed the molecular response mechanism of non-heading Chinese Cabbage to Iron Deficiency stress. Front Plant Sci. 2022; 13. 10.3389/fpls.2022.848424 PMC8964371 35371147

