# Supplementary material for: The activation of iron deficiency responses of grapevine rootstocks is dependent to the availability of the nitrogen forms
Source: BMC Plant Biol. 2024 Mar 26;24:218. doi: 10.1186/s12870-024-04906-y (PMC10964708; doi:10.1186/s12870-024-04906-y)
Supplement: Supplementary file 1 — Supplementary Material 1 [file 12870_2024_4906_MOESM1_ESM.docx]

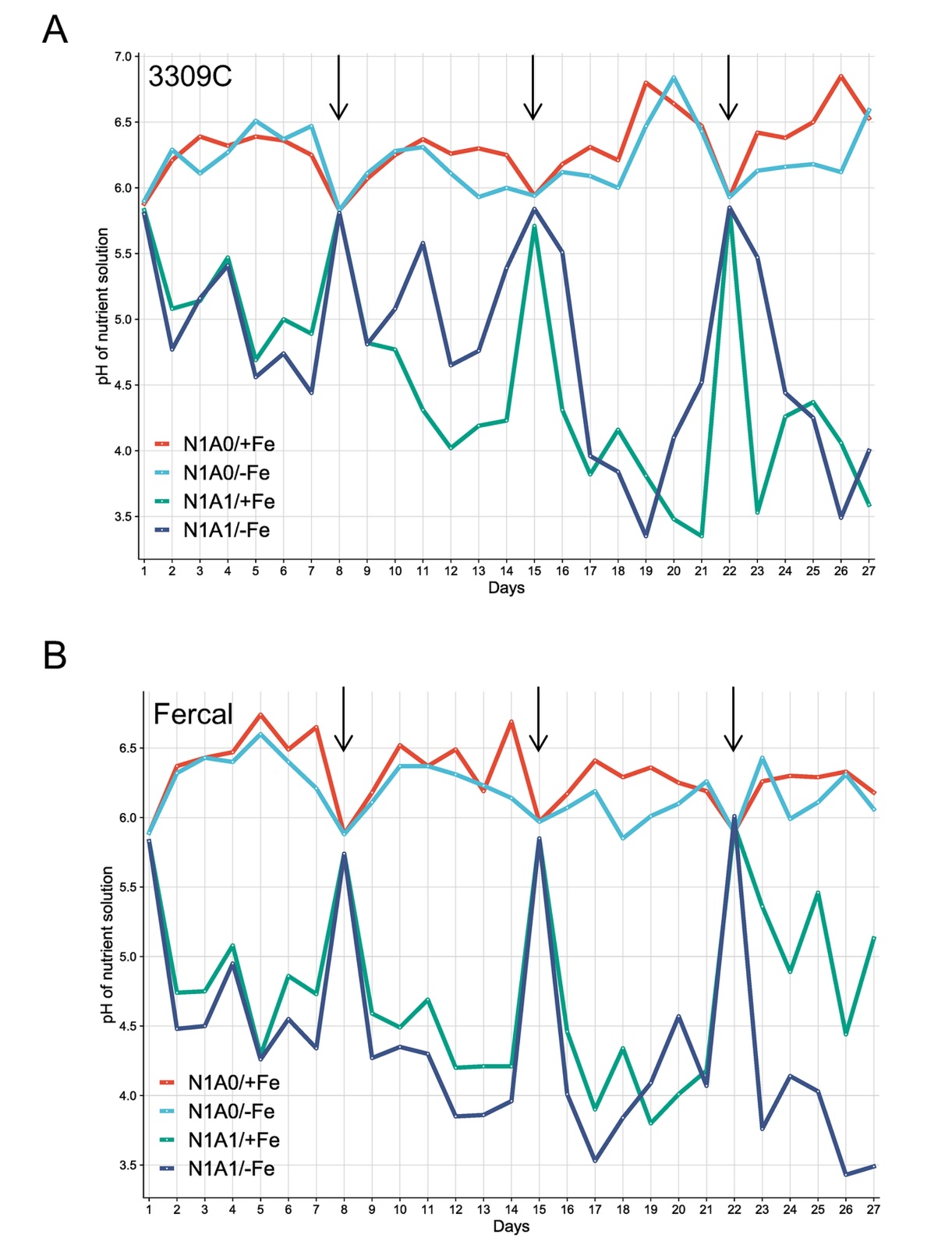
**Additional files**

**Figure S1.** Changes in the pH value of the nutrient solution of two grapevine rootstocks (A) 3309C and (B) Fercal grown in Fe-sufficient (+Fe) and Fe-deficient (−Fe) in interaction with two nitrogen forms (N1A0 – 100% nitrate; N1A1 – 50% nitrate, 50% ammonium). The nutrient solution was renewed one time per week during the experiment (arrow).

**Figure S2.** Relationships between chlorophyll concentration and (A) iron content; (B) modified chlorophyll absorption in reflectance index (MCARI) and (C) normalized difference vegetation index (NDVI), in young leaves of two grapevine rootstocks, 3309C, and Fercal grown in Fe-sufficient (+Fe) and Fe-deficient (−Fe) in interaction with two Nitrogen form.
